# The epidemiological signature of influenza B virus and its B/Victoria and B/Yamagata lineages in the 21^st^ century

**DOI:** 10.1371/journal.pone.0222381

**Published:** 2019-09-12

**Authors:** Saverio Caini, Gabriela Kusznierz, Verònica Vera Garate, Sonam Wangchuk, Binay Thapa, Francisco José de Paula Júnior, Walquiria Aparecida Ferreira de Almeida, Richard Njouom, Rodrigo A. Fasce, Patricia Bustos, Luzhao Feng, Zhibin Peng, Jenny Lara Araya, Alfredo Bruno, Doménica de Mora, Mónica Jeannette Barahona de Gámez, Richard Pebody, Maria Zambon, Rocio Higueros, Rudevelinda Rivera, Herman Kosasih, Maria Rita Castrucci, Antonino Bella, Hervé A. Kadjo, Coulibaly Daouda, Ainash Makusheva, Olga Bessonova, Sandra S. Chaves, Gideon O. Emukule, Jean-Michel Heraud, Norosoa H. Razanajatovo, Amal Barakat, Fatima El Falaki, Adam Meijer, Gé A. Donker, Q. Sue Huang, Tim Wood, Angel Balmaseda, Rakhee Palekar, Brechla Moreno Arévalo, Ana Paula Rodrigues, Raquel Guiomar, Vernon Jian Ming Lee, Li Wei Ang, Cheryl Cohen, Florette Treurnicht, Alla Mironenko, Olha Holubka, Joseph Bresee, Lynnette Brammer, Mai T. Q. Le, Phuong V. M. Hoang, Clotilde El Guerche-Séblain, John Paget, the Global Influenza B Study team

**Affiliations:** 1 Netherlands Institute for Health Services Research (Nivel), Utrecht, The Netherlands; 2 National Institute of Respiratory Diseases "Emilio Coni", Santa Fe, Argentina; 3 Royal Centre for Disease Control, Department of Public Health, Ministry of Health, Thimphu, Bhutan; 4 Ministry of Health, Department of Surveillance of Transmissible Diseases, Brasília/DF, Brazil; 5 Virology Department, Centre Pasteur of Cameroon, Yaoundé, Cameroon; 6 Sub-Department of Viral Diseases, Instituto de Salud Pública de Chile, Santiago, Chile; 7 Division of Infectious Diseases, Chinese Center for Disease Control and Prevention, Beijing, P.R. China; 8 National Influenza Center, Ministry of Health, San José, Costa Rica; 9 National Institute of Public Health Research (INSPI), National Reference Centre for Influenza and Other Respiratory Viruses, Guayaquil, Ecuador; 10 Agricultural University of Ecuador, Guayaquil, Ecuador; 11 National Influenza Center, Ministry of Health, San Salvador, El Salvador; 12 Public Health England, London, England, United Kingdom; 13 National Influenza Center, Ministry of Health, Guatemala City, Guatemala; 14 National Influenza Center, Ministry of Health, Tegucigalpa, Honduras; 15 US Naval Medical Research Unit No.2, Jakarta, Indonesia; 16 National Influenza Center, Department of Infectious Diseases, National Institute of Health, Rome, Italy; 17 Department of Infectious Diseases, National Institute of Health, Rome, Italy; 18 Department of Epidemic Virus, Institut Pasteur, Abidjan, Côte d'Ivoire; 19 Service of Epidemiological Diseases Surveillance, National Institute of Public Hygiene, Abidjan, Côte d'Ivoire; 20 National Center of Expertise, Committee of Public Health Protection, Ministry of Health, Astana, Kazakhstan; 21 National Center of Expertise, Committee of Public Health Protection, Ministry of Health, Uralsk City, Kazakhstan; 22 Influenza Division, Centers for Disease Control and Prevention, Atlanta, Georgia, United States of America; 23 Influenza Program, Centers for Disease Control and Prevention, Nairobi, Kenya; 24 National Influenza Center, Virology Unit, Institut Pasteur de Madagascar, Antananarivo, Madagascar; 25 National Influenza Center, Institut National d'Hygiène, Ministry of Health, Rabat, Morocco; 26 National Institute for Public Health and the Environment, Centre for Infectious Diseases Research, Diagnostics and Laboratory Surveillance, Bilthoven, The Netherlands; 27 Institute of Environmental Science and Research, Weillngton, New Zealand; 28 National Influenza Center, Ministry of Health, Managua, Nicaragua; 29 Pan American Health Organization, Washington, District of Columbia, United States of America; 30 National Influenza Center, IC Gorgas, Panama City, Panama; 31 Department of epidemiology, National Institute of Health Doutor Ricardo Jorge, Lisbon, Portugal; 32 National Influenza Reference Laboratory, National Institute of Health Doutor Ricardo Jorge, Lisbon, Portugal; 33 Public Health Group, Ministry of Health, Singapore, Singapore; 34 Centre for Respiratory Diseases and Meningitis, National Institute for Communicable Diseases of the National Health Laboratory Service, Johannesburg, South Africa; 35 School of Public Health, Faculty of Health Sciences, University of the Witwatersrand, Johannesburg, South Africa; 36 L.V.Gromashevsky Institute of Epidemiology and Infectious Diseases, National Academy of Medical Science of Ukraine, Department of Respiratory and other Viral Infections, Kyiv, Ukraine; 37 Influenza Division, National Center for Immunizations and Respiratory Diseases, Centers for Disease Control and Prevention, Atlanta, Georgia, United States of America; 38 National Institute of Hygiene and Epidemiology, Hanoi, Vietnam; 39 Global Vaccine Epidemiology and Modeling Department (VEM), Franchise Epidemiologist, Sanofi Pasteur, Lyon, France; Istituto Zooprofilattico Sperimentale delle Venezie, ITALY

## Abstract

We describe the epidemiological characteristics, pattern of circulation, and geographical distribution of influenza B viruses and its lineages using data from the Global Influenza B Study. We included over 1.8 million influenza cases occurred in thirty-one countries during 2000–2018. We calculated the proportion of cases caused by influenza B and its lineages; determined the timing of influenza A and B epidemics; compared the age distribution of B/Victoria and B/Yamagata cases; and evaluated the frequency of lineage-level mismatch for the trivalent vaccine. The median proportion of influenza cases caused by influenza B virus was 23.4%, with a tendency (borderline statistical significance, p = 0.060) to be higher in tropical vs. temperate countries. Influenza B was the dominant virus type in about one every seven seasons. In temperate countries, influenza B epidemics occurred on average three weeks later than influenza A epidemics; no consistent pattern emerged in the tropics. The two B lineages caused a comparable proportion of influenza B cases globally, however the B/Yamagata was more frequent in temperate countries, and the B/Victoria in the tropics (p = 0.048). B/Yamagata patients were significantly older than B/Victoria patients in almost all countries. A lineage-level vaccine mismatch was observed in over 40% of seasons in temperate countries and in 30% of seasons in the tropics. The type B virus caused a substantial proportion of influenza infections globally in the 21^st^ century, and its two virus lineages differed in terms of age and geographical distribution of patients. These findings will help inform health policy decisions aiming to reduce disease burden associated with seasonal influenza.

## Introduction

Influenza causes a major burden of disease on populations globally [1]. The impact of seasonal and pandemic influenza on population health in high-income countries has been described extensively [2]. In Europe, influenza ranks first among infectious diseases in terms of burden, accounting for 30% of the total disability-adjusted life years (DALYs) lost due to infectious diseases annually [3]. More recently, evidence has been accumulating that the burden of disease associated with influenza is high in low- and middle-income countries as well [4]. Type A viruses cause most influenza cases and are responsible for pandemics, but influenza B is an important cause of morbidity and mortality during interpandemic periods, and its prevention represents an important public health priority globally [5–6]. Influenza B viruses split into two antigenically distinct phylogenetic lineages (B/Victoria/2/87 representative, abbreviated B/Victoria, and B/Yamagata/16/88 representative, abbreviated B/Yamagata) in the early 1980s [7]. Whilst the circulation of the Victoria lineage was geographically limited to eastern Asia for most of the 1990s, the two lineages have co-circulated globally in the 21^st^ century [8–9].

The mainstay of influenza prevention is vaccination, aimed primarily at individuals who are at greater risk of developing complications when infected, like the elderly, pregnant women, and people with underlying medical conditions, and more recently at children to provide both direct protection to the children and indirect protection to the wider population [10]. Both the composition and period of administration of the vaccine are crucial to ensure adequate immunity against influenza; thus, investigating the timing of influenza epidemics and the patterns of circulation of the different influenza virus types, subtypes and lineages is of great importance from a public health perspective [11]. Until 2012, trivalent influenza vaccines (TIV) were available that contained only one type B influenza virus, belonging to either the B/Victoria or B/Yamagata lineage. Each year, the WHO issued recommendations for the B virus strain to be included in the TIV, but the frequency of lineage-level vaccine mismatch has been high (around 50%) [5]. This often resulted in unsatisfactory protection, considering the limited efficacy against the mismatched lineage [12], and substantial health impact [13]. Quadrivalent influenza vaccine (QIV), containing one B/Victoria and one B/Yamagata virus strain, were first approved by the US Food and Drug Administration (US-FDA) in 2012. This prompted the establishment of several studies that aimed to describe the epidemiology, clinical characteristics and burden of disease of influenza B in more depth, and to compare the cost-effectiveness of QIV vs. TIV in different settings and populations.

Despite significant advances in our understanding of influenza B [14], important knowledge gaps persist, in particular concerning the pattern of circulation and geographical distribution of B/Victoria and B/Yamagata viruses, and the age distribution of patients who are most often infected with either lineage. These aspects are of great importance, however, for instance in order to evaluate the cost-effectiveness of alternative influenza prevention strategies and thus enable more appropriate public health choices (e.g. around the choice of tri- and quadrivalent vaccines) [15]. In fact, while some reports have addressed this topic in single countries [16–22], a global overview is still lacking. Here, we conducted a global analysis of the epidemiology of influenza B virus and its lineages using the database of the Global Influenza B Study (GIBS).

## Methods

### The Global Influenza B Study

**Fig 1:**
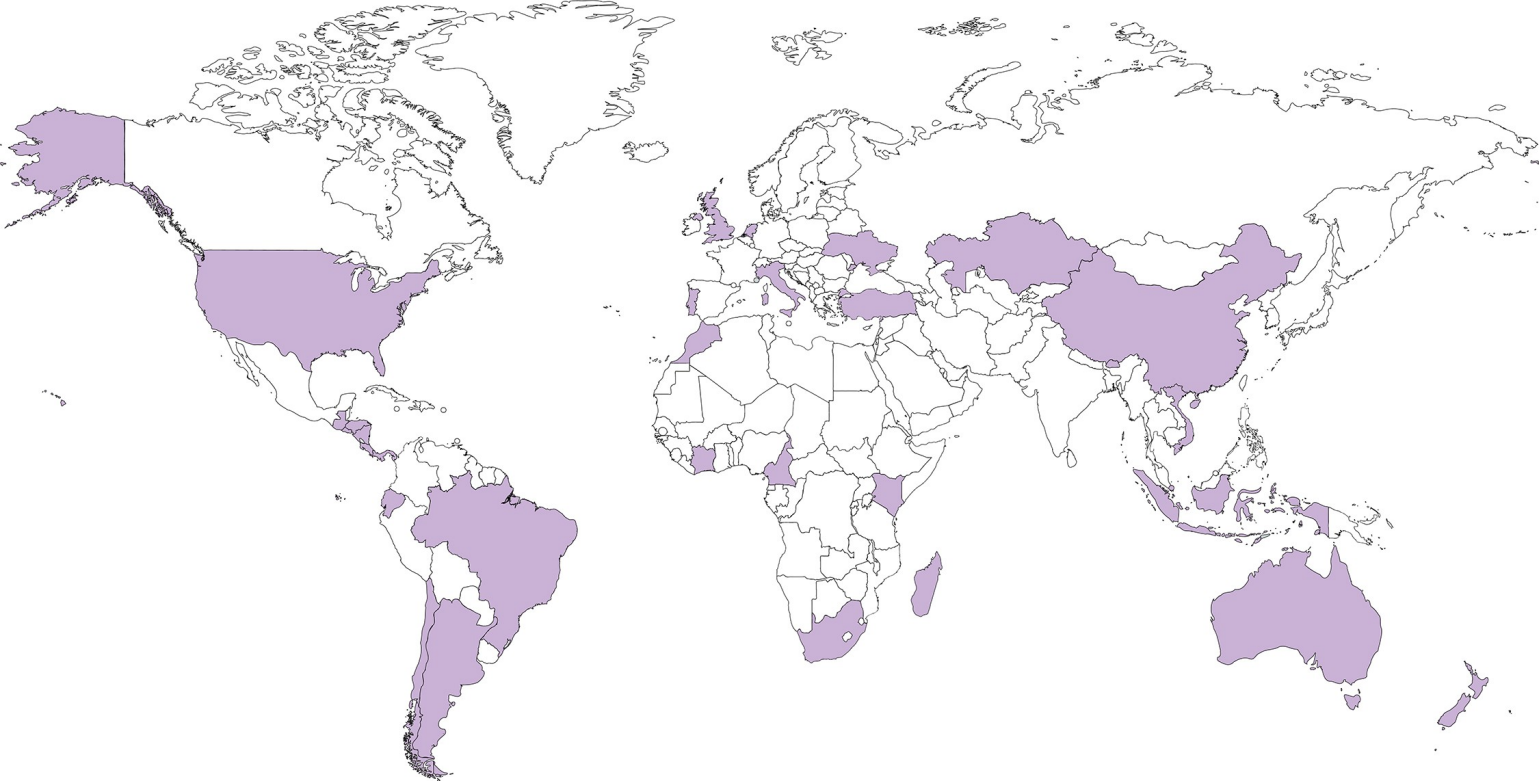
Countries participating in the Global Influenza B Study (GIBS), 2000–2018.

The rationale and methodology of the Global Influenza B Study has been described in detail elsewhere [14,23–24]. The GIBS database encompasses epidemiological and virological influenza surveillance data from thirty-one countries around the world (Fig 1). Participating countries were classified as located in the Northern hemisphere (n = 11), inter-tropical belt (n = 15), or Southern hemisphere (n = 5) based on the latitude of the country’s population centre [25]. Participating countries were required to make available influenza surveillance data for as many consecutive years as possible (from 2000 onwards). Data include the weekly number of laboratory-confirmed influenza cases broken down by virus type (A, B), type A subtype (H1N1, 2009 pandemic H1N1, H3N2, A not subtyped), and type B lineage (Victoria, Yamagata, B not characterized) and weekly influenza-like illness/acute respiratory infection rates (per 100,000 population or 100 consultations, depending on country). Information on age (exact age or age groups) was also available. In most participating countries, the influenza surveillance system covers the whole national territory, and a sample of influenza virus positive specimens or virus isolates is routinely sent to a WHO collaborating centre for further investigations. However, countries differ in other important aspects of their surveillance system, e.g. the clinical case definition in use, the distribution of outpatients and inpatients being sampled, and the availability of population denominators [14,24]. The GIBS database was initially assembled in 2013–14 by merging influenza surveillance data up to as late as December 2013 from thirty countries [14], but was updated for the purposes of this paper with data until 2015 to 2018 (depending on the country) for twenty-five countries. The Netherlands joined the project in 2018, bringing the total number of participating countries to thirty-one.

Data for China were provided separately for the Northern and Southern parts of the country, which were therefore entered separately in the analyses (for brevity, however, we will us the term “country” to refer to a whole country or a part of it henceforth). Brazil also provided data stratified by sub-national regions (i.e. five administrative regions: north, north-east, central-west, south, and south-east); however, geographically-stratified data were too sparse in some years, thus we opted to treat Brazil as a single country in the analysis.

### Statistical methods

Similar to previous GIBS publications [14,23–24], the unit of analysis was the “season”: this corresponded to the calendar year in tropical countries and countries located in the Southern hemisphere, and was defined as the period between the 27^th^ week of a year and the 26^th^ week of the following year for countries located in the Northern hemisphere. As previously explained [23], the purpose of this methodological approach is to give each “season” an equal weighting in the analysis, thus limiting the impact on results arising from any differences in reporting between countries (e.g. high- vs. low-resources countries) and over time within the same country (e.g. before vs. after the 2009 pandemic).

For each country, we included in the analyses the seasons with at least 20 weeks of reported data and at least 100 laboratory-confirmed influenza cases. In each season, we calculated the percentage of influenza cases that were due to influenza B. We then determined the percentage of cases that were caused by viruses belonging to the B/Victoria or B/Yamagata lineage: in this analysis, to increase the stability of the results, we only included (for each country) the subset of seasons with 50 or more characterized influenza B viruses. The non-parametric Kruskal-Wallis test was applied to compare the median proportion of influenza A and B cases (over all influenza cases) and B/Victoria and B/Yamagata cases (over all cases with characterized influenza B viruses) between countries based on their latitude (Northern hemisphere, inter-tropical belt, or Southern hemisphere) and population age structure (median age below 30 years, between 30 and 35 years, or above 35 years) [25].

We compared the timing of the primary peak of influenza A and B epidemics in each country using the EPIPOI software [26]. For this analysis, we excluded data from the 2009 season (2009–2010 in Northern hemisphere countries) because of the markedly atypical timing of the influenza A(H1N1)pdm09 pandemics. Time series were first standardized by dividing the weekly number of influenza cases by the maximum weekly number (per country by defined season). Next, a periodic annual function (PAF) was generated by summing up the annual, semi-annual and quarterly harmonics (obtained by Fourier decomposition). The timing of the primary peak (defined as the month where the PAF reaches its maximum value) was then compared for A and B epidemics in each country.

We aimed to evaluated the frequency of influenza B lineage-level vaccine mismatch, which was defined as a mismatch between the lineage that caused the majority (>50%) of influenza B cases in a given season and country, and the lineage included in the trivalent vaccine. Information on influenza vaccine formulation was retrieved from the WHO website [27]. The above definition is straightforward for temperate countries in the Northern and Southern hemisphere, but much less so for countries of the inter-tropical belt. While the WHO issues recommendations on the composition of influenza vaccines to be used in tropical countries [28], these countries often adopt either formulation based on local considerations or make no specific recommendations [29]. To cope with this and consider all possible scenarios (and also for consistency with previous GIBS publications [14]), the frequency of B lineage-level vaccine mismatch in tropical countries was calculated by assuming that: (i) all countries situated north of the equator use the northern hemisphere vaccine, and all countries south of the equator use the southern hemisphere vaccine formulation; (ii) all countries use the northern hemisphere vaccine formulation; or (iii) all countries use the southern hemisphere vaccine formulation.

The recent update of the GIBS database and the resulting increase in the number of cases with characterized influenza B viruses made it possible to compare the age distribution of influenza B/Victoria and B/Yamagata cases in a similar way to what had been done previously for influenza B vs. A (and its subtypes) [14,24]. This analysis was limited to countries for which information on the exact age was available for at least 50 B/Victoria and at least 50 B/Yamagata influenza cases over all seasons available. The virus-lineage-specific age distribution of influenza B cases was visualized, separately in each country, using histograms with 5-age-year-wide bars. Since the distributions were skewed to the right, we used the non-parametric Wilcoxon rank sum test to compare the median age of influenza B cases by virus lineage.

Statistical analyses were conducted using Stata software version 14 (StataCorp LP, College Station, TX, USA). All statistical tests were two-sided, and p-values were considered statistically significant when below 0.05.

## Results

The database of the Global Influenza B Study encompassed a total of 1.820.301 influenza cases between 2000 and 2018, of which 419,167 (23.0%) had type B influenza. Cases were unevenly distributed between countries, with USA and Australia contributing 54.1% and 25.3% of all influenza cases to the database, respectively. Information on age was not available for the USA; for the remaining countries, information on age was available for most (95.0%) influenza cases. After applying our exclusion criteria, 299 seasons with 100 or more reported influenza cases overall were included in the analysis, of which 110 were from Northern hemisphere countries, 131 from countries located in the inter-tropical belt, and 58 from Southern hemisphere countries. The median number of seasons per country was nine, ranging from three (for Turkey) to eighteen (for New Zealand). The number of overall and influenza B cases in each country and season included in the analysis, the proportion of influenza B cases of which the virus was characterized, and their breakdown into B/Victoria and B/Yamagata lineages, are provided in S1 Table.

### Frequency of influenza B

**Fig 2:**
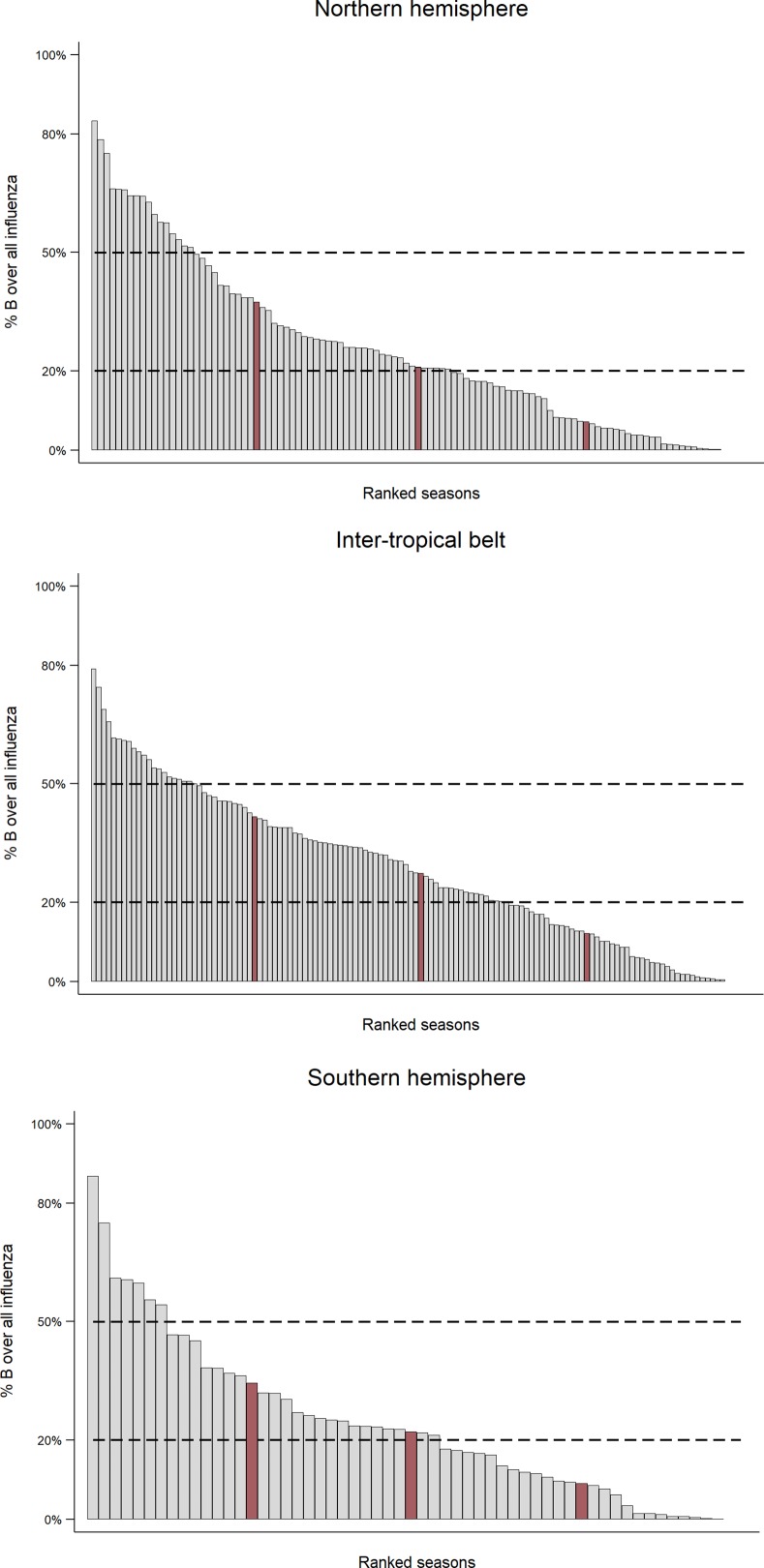
Distribution of influenza seasons by proportion of influenza B cases and geographical area (Inter-tropical belt, Northern hemisphere, Southern hemisphere). Red bars indicate 25^th^, 50^th^ (median) and 75^th^ percentiles. The Global Influenza B Study, 2000–2018.

Influenza B virus caused a median 23.4% (interquartile range [IQR] 9.3–38.9%) of reported influenza cases in a season. More specifically, the proportion of influenza cases caused by the B virus type was <20% in 118 seasons (42.3%), between 20% and 50% in 125 seasons (41.8%), and above 50% in 45 seasons (15.1%). The proportion of influenza B cases over all influenza cases reported in the season varied geographically (Fig 2): its median value tended to be higher (with borderline statistical significance, p = 0.060) in countries of the inter-tropical belt (27.4%, IQR 12.2–41.7%) compared to temperate countries of the Northern (21.0%, IQR 7.3–37.4%) and Southern (22.2%, IQR 9.1–34.5%) hemispheres. The proportion of seasons in which influenza B cases constituted between 20 and 50% or more than 50% of all reported influenza cases did not significantly differ (p = 0.275) between tropical countries (47.3% and 16.0%, respectively) and temperate countries of the two hemispheres (39.9% and 14.3%, respectively).

### Timing of influenza A and B epidemics

In countries of the Southern hemisphere, influenza A epidemics typically peaked in July-September, on average 1.1 month earlier than influenza B (August-September) (Table 1). Influenza epidemics also peaked in the winter months of Northern hemisphere countries, earlier for influenza A (January-February, except Ukraine, in March) than for influenza B (February-March, except England, in January), with an average difference of 0.6 months. The only exception was Bhutan (i.e. the southernmost countries among those situated in the Northern hemisphere), where both influenza A and B epidemics typically peaked in August. There was more variability in countries of the inter-tropical belt: the timing of the primary peak varied widely between countries and could take place in practically any month of the year, with a small difference in timing (0.3 months earlier on average) for the primary peak of influenza A and B epidemics.

**Table 1:** Typical timing of the peak of influenza A and B epidemics according to countries’ latitude. The Global Influenza B Study, 2000–2018.

Country	Latitude	Typical timing of influenza peak	
		A	B
**Southern hemisphere**			
New Zealand	41.8 S	Aug (1^st^ half)	Aug (2^nd^ half)
Chile	35.8 S	Jul (2^nd^ half)	Sep (2^nd^ half)
Argentina (Santa Fe)	31.4 S	Aug (1^st^ half)	Sep (2^nd^ half)
South Africa	29.0 S	Jul (1^st^ half)	Sep (2^nd^ half)
Australia	25.8 S	Sep (1^st^ half)	Sep (1^st^ half)
**Inter-tropical belt**			
Madagascar	19.4 S	Feb (1^st^ half)	Mar (1^st^ half)
Brazil	10.8 S	Jun (2^nd^ half)	Oct (1^st^ half)
Ecuador	2.0 S	May (1^st^ half)	Jul (1^st^ half)
Indonesia	1.7 S	Feb (1^st^ half)	Apr (2^nd^ half)
Kenya	0.4 S	Jul (2^nd^ half)	Mar (2^nd^ half)
Singapore	1.2 N	Jun (1^st^ half)	May (2^nd^ half)
Cameroon	5.7 N	Nov (1^st^ half)	Nov (1^st^ half)
Ivory Coast	7.6 N	Nov (2^nd^ half)	Oct (1^st^ half)
Panama	8.6 N	Jun (2^nd^ half)	Jul (2^nd^ half)
Costa Rica	10.0 N	Dec (2^nd^ half)	Oct (1^st^ half)
Nicaragua	12.9 N	Nov (1^st^ half)	Aug (2^nd^ half)
El Salvador	13.8 N	Jun (2^nd^ half)	Jul (1^st^ half)
Honduras	14.8 N	Nov (1^st^ half)	Jul (1^st^ half)
Guatemala	15.7 N	Mar (2^nd^ half)	Sep (1^st^ half)
Viet Nam	16.7 N	Aug (1^st^ half)	Dec (1^st^ half)
**Northern hemisphere**			
Bhutan	27.4 N	Aug (2^nd^ half)	Aug (2^nd^ half)
China South	31.1 N	Feb (2^nd^ half)	Mar (2^nd^ half)
Morocco	32.0 N	Jan (2^nd^ half)	Feb (1^st^ half)
Turkey	39.0 N	Feb (1^st^ half)	Feb (2^nd^ half)
Portugal	39.3 N	Jan (2^nd^ half)	Feb (1^st^ half)
China North	39.5 N	Jan (2^nd^ half)	Mar (1^st^ half)
Italy	42.9 N	Feb (2^nd^ half)	Feb (2^nd^ half)
USA	45.6 N	Feb (1^st^ half)	Mar (2^nd^ half)
Kazakhstan	48.0 N	Feb (2^nd^ half)	Feb (2^nd^ half)
Ukraine	49.1 N	Mar (1^st^ half)	Mar (1^st^ half)
Netherlands	52.3 N	Feb (1^st^ half)	Mar (1^st^ half)
England	52.3 N	Jan (2^nd^ half)	Jan (2^nd^ half)

### Frequency of B/Victoria and B/Yamagata lineages viruses

There were 84 seasons where the virus lineage was determined for 50 or more influenza B cases (out of a total 299 seasons, 28.1%) and this data covered 18 countries (out of 31). In these seasons, the B/Victoria and B/Yamagata lineage viruses caused a similar range of proportions of influenza B cases. More precisely, the median proportion of influenza B cases reported in a season that were caused by B/Victoria lineage viruses was 46.0% (IQR 5.3–89.6%). The range of the proportion of B/Victoria over all influenza B cases was 0%-100%, meaning that there were seasons in which all influenza B cases were caused by either virus lineage. Co-circulation of the two lineages was frequent: in 27 (32.1%) of 84 seasons, both lineages accounted for at least 20% of influenza B cases, while the B/Victoria and B/Yamagata lineages caused over 80% of all influenza B cases in 27 (32.1%) and 30 (35.8%) seasons, respectively.

**Fig 3:**
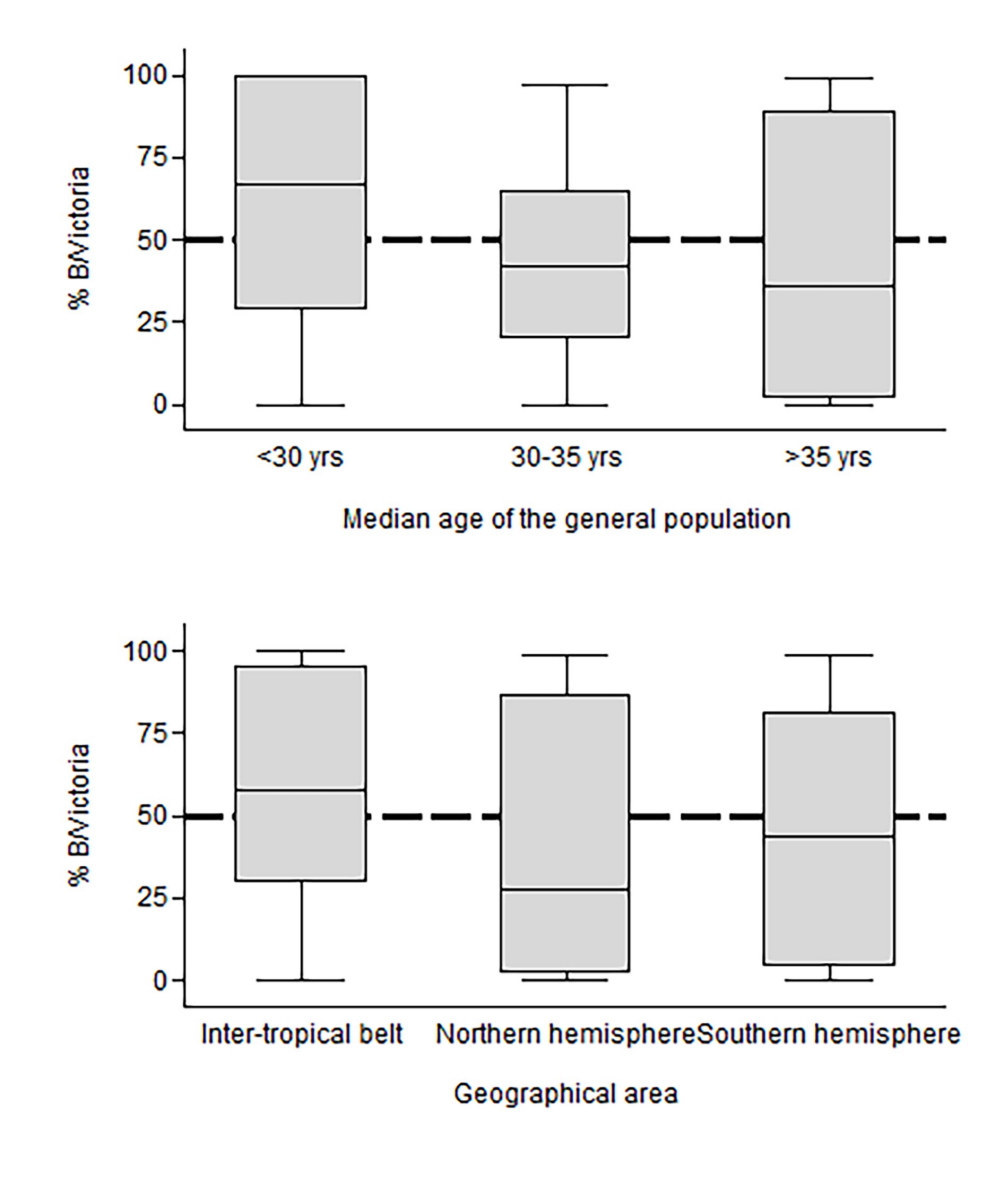
Proportion of influenza B cases in a season that were caused by the B/Victoria lineage viruses, according to countries median age (top) and geographical area (bottom). The Global Influenza B Study, 2000–2018.

The proportion of either lineage over all influenza B cases varied according to the age structure of countries, and consequently, according to the countries’ latitude as well (Fig 3). The proportion of influenza B cases caused by the B/Victoria lineage in a given season varied widely (ranging from 0% to nearly 100%) also in the analyses stratified by the country’s age structure and latitude. However, the B/Victoria lineage was more frequent (p-value 0.049) in countries with a median age below 30 years (median proportion over all influenza B cases = 67.0%) compared to countries with a median age was 30–35 years (42.0%) or above 35 years (36.2%). Likewise, the median proportion of B/Victoria over all characterized influenza B cases in a season was higher in countries of the inter-tropical belt (57.5%) compared to temperate countries of the Northern (27.9%) and the Southern hemisphere (43.8%) (p-value = 0.048).

### Age distribution of B/Victoria and B/Yamagata influenza cases

In Table 2, we compared the median age of influenza B cases according to virus lineage. In Australia, B/Victoria cases were non-significantly older than B/Yamagata cases: the median age was 11 vs. 8 years, respectively. In all other countries, B/Yamagata cases were older than B/Victoria cases, and the difference between the median age was always significant except in Madagascar (p-value 0.248) and in Ukraine (p-value 0.082).

**Table 2:** Age distribution of influenza B/Victoria and B/Yamagata patients by country (sorted by median age of the general population). The Global Influenza B Study, 2000–2018.

Country ^(a)^	Median age general population	No. influenza B cases		Median age (IQR)		
		Victoria	Yamagata	Victoria	Yamagata	p-value ^(b)^
Madagascar	19.7	787	405	7 (4–16)	8 (3–17)	0.248
Ivory Coast	20.9	240	153	3 (1–8)	5 (2–32)	0.002
South Africa	27.1	61	102	3 (1–29)	29 (1–54)	0.003
Indonesia	30.2	422	235	10 (6–20)	25 (7–36)	<0.001
Turkey	30.9	98	339	12 (6–22)	34 (11–48)	<0.001
Chile	34.4	1351	1950	8 (3–18)	14 (6–21)	<0.001
Singapore	34.6	413	921	15 (8–32)	35 (12–51)	<0.001
New Zealand	37.9	232	1036	15 (7–33)	35 (11–52)	<0.001
Australia	38.7	648	276	11 (5–23)	8 (3–36)	0.468
England	40.5	478	877	16 (8–29)	40 (17–53)	<0.001
Ukraine	40.6	109	186	13 (8–19)	15 (7–32)	0.082
Portugal	42.2	57	183	19 (13–36)	46 (34–57)	<0.001
Netherlands	42.6	354	773	19 (7–36)	46 (26–58)	<0.001
Italy	45.5	68	510	10 (6–19)	12 (6–47)	0.009

IQR: inter-quartile range

^(a)^ Countries were included if information on exact age was available for ≥50 B Victoria and ≥50 B Yamagata influenza cases.

^(b)^ Wilcoxon rank-sum test for comparison of medians

The visual inspection of the graphs revealed a fairly consistent pattern in the age distribution of B/Victoria and B/Yamagata cases (S1 File). In most countries, influenza B/Victoria cases tended to be distributed according to their age along a unimodal curve, with a peak below 10 years of age, while the age distribution of B/Yamagata cases frequently followed a bimodal curve, with an earlier, larger peak below 10 years of age, and a smaller, yet discernible peak at older age, mostly between 25 and 50 years of age. The adult age peak of B/Yamagata cases was barely noticeable in countries where the majority of inhabitants are younger than 25 years (e.g. Madagascar and Ivory Coast), and became increasingly evident as the median age of the country population increased. A partial exception was Portugal, where the peak in the age distribution of B/Victoria cases was between 10 and 20 years of age, and B/Yamagata distributed along a unimodal curve peaking at above 50 years of age.

### Frequency of lineage-level influenza B trivalent vaccine mismatches

The proportion of influenza B lineage-level vaccine mismatch was 54.2% in countries in the Southern hemisphere, and 42.9% in countries of the Northern hemisphere. For countries in the inter-tropical belt, the proportion of seasons with a lineage-level vaccine mismatch was 29.6% in the scenario in which these countries use the vaccine composition recommended for the hemisphere they are situated in; 40.7% assuming all these countries used the recommended vaccine composition for the Northern hemisphere; and 22.2% if all countries used the recommended vaccine composition for the Southern hemisphere.

## Discussion

Our analysis found that influenza B virus was responsible for nearly one fourth of all influenza cases in an average season between 2000 and 2018. More specifically, influenza B virus accounted for over 20% of all influenza cases in more than half of the seasons, and was the dominant virus type in about one out of every seven seasons. This proportion varied geographically, as the proportion of influenza B over all influenza cases tended to be higher on average in tropical countries compared to countries of the Northern and Southern hemispheres. These findings were broadly in line with reports from countries around the world [5,9,20–21,30–31] and with GIBS data published in 2015 [14], with moderate discrepancies (less than 5%) being most likely linked to the variability of the time span covered by different studies. Influenza B epidemics tended to peak on average three weeks later than influenza A epidemics during the winter period in temperate countries of both hemispheres, which is consistent with previous country reports [21,30,32–34]. The timing of influenza A and B epidemics was different in tropical countries where they were highly heterogeneous and showed no consistent pattern in the timing of the different epidemics. Influenza viruses exhibit differential ability to induce temporary immunity against re-infection with other viruses in experimental settings [35]. This “viral hierarchy” may help explain the differences in timing of epidemics caused by different virus types and subtypes where the weather conditions that are most favourable to the occurrence of influenza epidemics (e.g. cold-dry) last for only a limited number of weeks each year.

The study of patterns of circulation of B/Victoria and B/Yamagata lineages revealed two main findings. Firstly, the proportion of B cases caused by either lineage in a given season ranged between 0% and nearly 100% in each geographical area (i.e. anywhere around the world seasons occurred where all influenza B cases were caused by either the B/Victoria or the B/Yamagata lineage), which emphasizes the challenges faced when trying to predict which lineage will cause most influenza B cases next season. This helps explain the very high frequency of lineage-level vaccine mismatch for the TIV which was around 50% (i.e. close to the scenario in which the choice of the vaccine lineage is made at random) in temperate countries of the two hemispheres, which has been reported in the past [30,36–37].

The second important finding that emerges from the B/Victoria-B/Yamagata lineage analysis is that whilst the two lineages caused a comparable proportion of influenza B cases globally during the study period, their distribution varied geographically, as B/Victoria was relatively more frequent in tropical countries, while B/Yamagata was more frequent in temperate climate countries of the Southern and Northern hemispheres. The reason for this pattern of circulation of the two lineages is not clear. One possibility is that the survival and transmissibility of B/Victoria and B/Yamagata viruses is differentially affected by “cold-dry” and “humid-rainy” environmental conditions that drive the occurrence of influenza epidemics in temperate and tropical countries, respectively [38]. This hypothesis does not seem, however, to be supported by the available evidence, which suggests instead that influenza viruses of different types and subtypes (e.g. A(H3N2), A(H1N1)pdm09, and B) are similarly affected by weather conditions [39]. Based on our findings on the differential age distribution of B/Victoria and B/Yamagata infected cases, a more likely explanation for the unequal geographical distribution of the two B lineages lies is in the diverse demographic structure of world countries, with those located around the tropics having a lower median age, on average, than those in temperate climates.

In this regard, our study showed consistently at a global level (i.e. across countries that differ greatly under many aspects, including the type of influenza surveillance system) that B/Yamagata cases are on average older than B/Victoria cases, and in particular, that the former virus lineage infects adult individuals (≥25 years) more frequently than the latter. The only exception (i.e. the only country in which B/Victoria cases were older than B/Yamagata cases, albeit not significantly) was Australia. The reason for this divergent pattern is not clear; however, since the majority of B/Yamagata cases in the database from Australia occurred in 2008 (see S1 Table), this finding might be driven by a single season with an unusual age distribution of B/Yamagata patients. Importantly, our findings support earlier work showing that each influenza A subtype (H3N2, pre-2009-pandemic H1N1, and H1N1pdm09) tends to affect a different age group [24]. A recent study has shown that the B/Victoria viruses antigenic drift parallels that of A(H3N2), with limited cross-reactivity between phylogenetic clusters compared to B/Yamagata (which, in contrast, resembled A(H1N1) under this regard, with multiple variants circulating and greater levels of antigenic cross-reactivity) [40]. According to these observations, one would expect re-infection among adult individuals to occur more frequently for B/Victoria rather than B/Yamagata, but the observed pattern runs counter to this expectation. Vijaykrishna and colleagues suggested that the observed differential age distribution may be explained by either the higher effective reproductive number of B/Victoria; a broader response against B/Victoria viruses in older people; and/or differences in the prevalence of receptor binding molecules that help B/Victoria and B/Yamagata viruses infect respiratory tract cells in young children and adults [40]. Another hypothesis is that the bimodal age distribution of B/Yamagata cases may be the result of a shorter duration of acquired immunity developed after infection with B/Yamagata vs. B/Victoria viruses [41]. Finally, antigenic original sin (i.e. preferential response to influenza B viruses encountered in the past [42]) may also play a role. If this were true, the mean age of patients infected with either B lineage would be expected to drift over time: this has occurred in Australia between 2008–2011 [40], but we were unable to replicate these results in the GIBS database (results not shown).

These findings have important implications for assessing the impact of influenza B viruses in a population each season. Knowing early in the season (through surveillance activities) which influenza B lineage virus is circulating will allow one to predict which age groups will be more affected, with B/Yamagata seasons being generally characterized by more adults and elderly people being infected and, therefore, by a greater burden of disease compared to B/Victoria seasons. This appears to have been the case during the 2017/18 season in Europe, when B/Yamagata was dominant (and there was a lineage-level vaccine mismatch) and a high burden of influenza B was observed in many European countries [43].

We analyzed influenza surveillance data from around the world to determine the epidemiology of influenza B virus and its lineages in the 21^st^ century. We have enriched the analysis of an earlier GIBS paper [14] by including more countries (31 vs. 26) and additional years (data to up to 2018): the overall number of reports analysed is now twice as large (>1.8 million *vs*. 935,000 cases). This allowed us to conduct a comprehensive comparative analysis of the epidemiological characteristics of B/Victoria and B/Yamagata virus lineages, thus achieving a better understanding of the epidemiology of influenza B globally. The most important limitation of our study is that it relies on data that were collected within national surveillance systems rather than for research purposes. Countries participating in GIBS inevitably differ in terms of a number of important characteristics, including percentage of out- and in-patients included, number of years with data and average number of influenza cases reported per year, proportion of influenza B viruses that were characterized availability of information on age of these cases and sampling strategies across age groups. In each individual country, changes in the aforementioned characteristics may have occurred over time, for instance before and after the 2009 pandemic. Another limitation of our study is the uneven distribution of cases among countries and seasons, which is partly due to differences among countries in terms of population, the intensity of surveillance activities, and the coverage of each country’s influenza surveillance system. Throughout this paper, we have used statistical methods that were intended to minimize the impact of this variability on the results. These include the use of the ‘season’ as a unit of analysis for the comparison of the frequency of B influenza across countries, the standardization offered by the EPIPOI software in the analysis of temporal patterns of influenza epidemics, and the use of medians to compare the age distribution of influenza cases between countries. However, we acknowledge that these sources of variability may have affected our findings to an extent that is difficult to quantify precisely, and a further improvement in the harmonization of influenza surveillance data remains desirable as it would allow researchers to conduct more detailed and in-depth analyses and obtain more reliable results.

In conclusion, our study provides important new information that describes the epidemiology of influenza B viruses and its lineages during the start of the 21^st^ century. We showed that influenza B virus caused nearly one fourth (on average) of all influenza cases occurring annually in the world during 2000–2018, and that both lineages accounted each for a comparable proportion of all influenza B cases globally. We also observed that cases infected with the B/Victoria or B/Yamagata lineages differed consistently (i.e. in a similar fashion across countries) in terms of their age distribution, and suggested that this may help explain the uneven geographical distribution of the two lineages globally. Finally, we confirmed that the TIV suffers from a high frequency of lineage-level mismatch, especially in temperate countries of the two hemispheres. While expanding our knowledge of the epidemiology and pattern of circulation of influenza B virus, these findings will help inform health policy decisions aiming to reduce disease burden associated with seasonal influenza locally, regionally and globally.

## Supporting information

S1 Table Laboratory-confirmed influenza cases reported in each country and season, and proportion of those caused by the influenza B virus type (overall and by lineage). **The Global Influenza B Study, 2000–2018**.
^(a)^ A season was defined as the period between the 27th week of a given year and the 26th week of the following year for countries in the Northern hemisphere, and as the period between the first and last week of a given year for other countries.
^(b)^ Seasons with fewer than 100 influenza cases were not included.
^(c)^ Number of influenza B cases in each season, and proportion of influenza B over all influenza cases in the same season.
^(d)^ Number of influenza B cases for which the virus lineage was characterized, and proportion over all influenza B cases in the same season.
^(e)^ Number of influenza B cases caused by viruses belonging to the Victoria and Yamagata lineages, and proportion over all characterized influenza B viruses in the same season. Not reported in seasons with fewer than 50 characterized influenza B cases.
(DOCX)

S1 File Age distribution of laboratory-confirmed influenza patients infected with type B viruses belonging to the Victoria or Yamagata lineage. Countries which provided information on exact age for ≥50 B Victoria and ≥50 B Yamagata influenza for the whole study period cases were included. Countries were ordered according to the median age of the general population. The Global Influenza B Study, 2000–2018.
(PDF)

S2 File Contact information of data owners. (DOC)
