# Supplementary material for: The epidemiological signature of influenza B virus and its B/Victoria and B/Yamagata lineages in the 21st century
Source: PLoS One. 2019 Sep 12;14(9):e0222381. doi: 10.1371/journal.pone.0222381 (PMC6742362; doi:10.1371/journal.pone.0222381)
Supplement: S1 Table — The Global Influenza B Study, 2000–2018. (a) A season was defined as the period between the 27th week of a given year and the 26th week of the following year for countries in the Northern hemisphere, and as the period between the first and last week of a given year for other countries. (b) Seasons with fewer than 100 influenza cases were not included. (c) Number of influenza B cases in each season, and proportion of influenza B over all influenza cases in the same season. (d) Number of influenza B cases for which the virus lineage was characterized, and proportion over all influenza B cases in the same season. (e) Number of influenza B cases caused by viruses belonging to the Victoria and Yamagata lineages, and proportion over all characterized influenza B viruses in the same season. Not reported in seasons with fewer than 50 characterized influenza B cases. (DOCX) [file pone.0222381.s001.docx]

| **Country** | **Season ^(a)^** | **Influenza notifications (n) ^(b)^** | **Influenza B notifications (n, %) ^(c)^** | **Lineage characterization (n, %) ^(d)^** | **Influenza B lineage (n, %) ^(e)^** | |
| --- | --- | --- | --- | --- | --- | --- |
|  |  |  |  |  | **Victoria** | **Yamagata** |
| Argentina | 2009 | 240 | 0 (0.0%) | - | - | - |
| Argentina | 2011 | 260 | 0 (0.0%) | - | - | - |
| Argentina | 2012 | 337 | 108 (32.0%) | 108 (100.0%) | 105 (97.2%) | 3 (2.8%) |
| Argentina | 2013 | 210 | 3 (1.4%) | 1 (33.3%) | - | - |
| Argentina | 2014 | 100 | 75 (75.0%) | 62 (82.7%) | 25 (40.3%) | 37 (59.7%) |
| Argentina | 2016 | 165 | 27 (16.4%) | 11 (40.7%) | - | - |
| Australia | 2001 | 1011 | 117 (11.6%) | 0 (0%) | - | - |
| Australia | 2002 | 3257 | 810 (24.9%) | 0 (0%) | - | - |
| Australia | 2003 | 2935 | 101 (3.4%) | 1 (1%) | - | - |
| Australia | 2004 | 1867 | 325 (17.4%) | 2 (0.6%) | - | - |
| Australia | 2005 | 4110 | 921 (22.4%) | 1 (0.1%) | - | - |
| Australia | 2006 | 3123 | 819 (26.2%) | 2 (0.2%) | - | - |
| Australia | 2007 | 9908 | 939 (9.5%) | 16 (1.7%) | - | - |
| Australia | 2008 | 9049 | 5024 (55.5%) | 390 (7.8%) | 154 (39.5%) | 236 (60.5%) |
| Australia | 2009 | 58865 | 473 (0.8%) | 0 (0%) | - | - |
| Australia | 2010 | 13270 | 1287 (9.7%) | 49 (3.8%) | - | - |
| Australia | 2011 | 27003 | 7293 (27.0%) | 189 (2.6%) | 185 (97.9%) | 4 (2.1%) |
| Australia | 2012 | 44363 | 10499 (23.7%) | 274 (2.6%) | 255 (93.1%) | 19 (6.9%) |
| Australia | 2013 | 27560 | 10200 (37.0%) | 0 (0%) | - | - |
| Australia | 2014 | 66335 | 7940 (12.0%) | 0 (0%) | - | - |
| Australia | 2015 | 99228 | 60212 (60.7%) | 0 (0%) | - | - |
| Australia | 2016 | 89138 | 9594 (10.8%) | 0 (0%) | - | - |
| Bhutan | 2009-10 | 424 | 87 (20.5%) | 0 (0%) | - | - |
| Bhutan | 2010-11 | 313 | 74 (23.6%) | 0 (0%) | - | - |
| Bhutan | 2011-12 | 165 | 95 (57.6%) | 0 (0%) | - | - |
| Bhutan | 2012-13 | 168 | 48 (28.6%) | 0 (0%) | - | - |
| Bhutan | 2015-16 | 132 | 55 (41.7%) | 9 (16.4%) | - | - |
| Bhutan | 2016-17 | 169 | 76 (45.0%) | 22 (28.9%) | - | - |
| Brazil | 2004 | 196 | 26 (13.3%) | 0 (0%) | - | - |
| Brazil | 2005 | 118 | 17 (14.4%) | 0 (0%) | - | - |
| Brazil | 2006 | 245 | 53 (21.6%) | 0 (0%) | - | - |
| Brazil | 2007 | 273 | 33 (12.1%) | 0 (0%) | - | - |
| Brazil | 2008 | 306 | 126 (41.2%) | 0 (0%) | - | - |
| Brazil | 2009 | 646 | 131 (20.3%) | 0 (0%) | - | - |
| Brazil | 2010 | 466 | 213 (45.7%) | 0 (0%) | - | - |
| Brazil | 2011 | 571 | 195 (34.2%) | 0 (0%) | - | - |
| Brazil | 2012 | 461 | 89 (19.3%) | 0 (0%) | - | - |
| Brazil | 2013 | 2009 | 727 (36.2%) | 0 (0%) | - | - |
| Brazil | 2014 | 1990 | 473 (23.8%) | 0 (0%) | - | - |
| Brazil | 2015 | 1860 | 667 (35.9%) | 0 (0%) | - | - |
| Brazil | 2016 | 2509 | 766 (30.5%) | 0 (0%) | - | - |
| Cameroon | 2009 | 127 | 1 (0.8%) | 0 (0%) | - | - |
| Cameroon | 2011 | 167 | 103 (61.7%) | 0 (0%) | - | - |
| Cameroon | 2012 | 180 | 29 (16.1%) | 0 (0%) | - | - |
| Cameroon | 2013 | 191 | 75 (39.3%) | 0 (0%) | - | - |
| Chile | 2008 | 700 | 54 (7.7%) | 9 (16.7%) | - | - |
| Chile | 2009 | 4123 | 52 (1.3%) | 0 (0%) | - | - |
| Chile | 2010 | 3076 | 418 (13.6%) | 29 (6.9%) | - | - |
| Chile | 2011 | 1162 | 17 (1.5%) | 7 (41.2%) | - | - |
| Chile | 2012 | 2101 | 638 (30.4%) | 601 (94.2%) | 195 (32.4%) | 406 (67.6%) |
| Chile | 2013 | 2602 | 597 (22.9%) | 525 (87.9%) | 269 (51.2%) | 256 (48.8%) |
| Chile | 2014 | 1882 | 315 (16.7%) | 279 (88.6%) | 12 (4.3%) | 267 (95.7%) |
| Chile | 2015 | 2246 | 565 (25.2%) | 505 (89.4%) | 221 (43.8%) | 284 (56.2%) |
| Chile | 2016 | 3727 | 876 (23.5%) | 708 (80.8%) | 571 (80.6%) | 137 (19.4%) |
| Chile | 2017 | 3610 | 821 (22.7%) | 638 (77.7%) | 47 (7.4%) | 591 (92.6%) |
| China North | 2005-06 | 1455 | 234 (16.1%) | 18 (7.7%) | - | - |
| China North | 2006-07 | 1899 | 332 (17.5%) | 331 (99.7%) | 278 (84.0%) | 53 (16.0%) |
| China North | 2007-08 | 1822 | 1206 (66.2%) | 1151 (95.4%) | 244 (21.2%) | 907 (78.8%) |
| China North | 2008-09 | 2153 | 391 (18.2%) | 309 (79%) | 278 (90.0%) | 31 (10.0%) |
| China North | 2009-10 | 35519 | 7486 (21.1%) | 1481 (19.8%) | 1411 (95.3%) | 70 (4.7%) |
| China North | 2010-11 | 9669 | 1400 (14.5%) | 460 (32.9%) | 75 (16.3%) | 385 (83.7%) |
| China North | 2011-12 | 10959 | 8615 (78.6%) | 4264 (49.5%) | 3016 (70.7%) | 1248 (29.3%) |
| China South | 2006-07 | 2764 | 537 (19.4%) | 502 (93.5%) | 190 (37.8%) | 312 (62.2%) |
| China South | 2007-08 | 2800 | 1671 (59.7%) | 1495 (89.5%) | 516 (34.5%) | 979 (65.5%) |
| China South | 2008-09 | 3568 | 1122 (31.4%) | 995 (88.7%) | 831 (83.5%) | 164 (16.5%) |
| China South | 2009-10 | 65839 | 13696 (20.8%) | 3127 (22.8%) | 2460 (78.7%) | 667 (21.3%) |
| China South | 2010-11 | 18479 | 4784 (25.9%) | 1559 (32.6%) | 817 (52.4%) | 742 (47.6%) |
| China South | 2011-12 | 19778 | 12725 (64.3%) | 6412 (50.4%) | 5724 (89.3%) | 688 (10.7%) |
| Costa Rica | 2009 | 3898 | 80 (2.1%) | 0 (0%) | - | - |
| Costa Rica | 2010 | 1441 | 176 (12.2%) | 0 (0%) | - | - |
| Costa Rica | 2011 | 194 | 27 (13.9%) | 0 (0%) | - | - |
| Costa Rica | 2012 | 444 | 166 (37.4%) | 0 (0%) | - | - |
| Costa Rica | 2013 | 550 | 10 (1.8%) | 0 (0%) | - | - |
| Costa Rica | 2014 | 518 | 242 (46.7%) | 0 (0%) | - | - |
| Costa Rica | 2015 | 265 | 0 (0.0%) | - | - | - |
| Costa Rica | 2016 | 459 | 28 (6.1%) | 0 (0%) | - | - |
| Costa Rica | 2017 | 591 | 231 (39.1%) | 0 (0%) | - | - |
| Ecuador | 2011 | 466 | 0 (0.0%) | - | - | - |
| Ecuador | 2012 | 410 | 185 (45.1%) | 0 (0%) | - | - |
| Ecuador | 2013 | 838 | 73 (8.7%) | 0 (0%) | - | - |
| Ecuador | 2014 | 158 | 72 (45.6%) | 13 (18.1%) | - | - |
| Ecuador | 2015 | 143 | 32 (22.4%) | 5 (15.6%) | - | - |
| Ecuador | 2016 | 966 | 92 (9.5%) | 42 (45.7%) | - | - |
| El Salvador | 2009 | 1098 | 18 (1.6%) | 0 (0%) | - | - |
| El Salvador | 2010 | 352 | 121 (34.4%) | 0 (0%) | - | - |
| El Salvador | 2011 | 202 | 101 (50.0%) | 0 (0%) | - | - |
| El Salvador | 2012 | 421 | 198 (47.0%) | 0 (0%) | - | - |
| El Salvador | 2013 | 234 | 3 (1.3%) | 0 (0%) | - | - |
| El Salvador | 2016 | 241 | 21 (8.7%) | 14 (66.7%) | - | - |
| El Salvador | 2017 | 288 | 49 (17.0%) | 45 (91.8%) | - | - |
| England | 2003-04 | 249 | 0 (0.0%) | - | - | - |
| England | 2004-05 | 145 | 22 (15.2%) | 2 (9.1%) | - | - |
| England | 2005-06 | 299 | 192 (64.2%) | 34 (17.7%) | - | - |
| England | 2006-07 | 456 | 8 (1.8%) | 2 (25%) | - | - |
| England | 2007-08 | 331 | 84 (25.4%) | 24 (28.6%) | - | - |
| England | 2008-09 | 640 | 52 (8.1%) | 4 (7.7%) | - | - |
| England | 2009-10 | 1338 | 11 (0.8%) | 5 (45.5%) | - | - |
| England | 2010-11 | 1679 | 661 (39.4%) | 321 (48.6%) | 300 (93.5%) | 21 (6.5%) |
| England | 2011-12 | 347 | 66 (19.0%) | 23 (34.8%) | - | - |
| England | 2012-13 | 764 | 470 (61.5%) | 204 (43.4%) | 20 (9.8%) | 184 (90.2%) |
| England | 2013-14 | 247 | 9 (3.6%) | 6 (66.7%) | - | - |
| England | 2014-15 | 608 | 134 (22.0%) | 50 (37.3%) | 4 (8.0%) | 46 (92.0%) |
| England | 2015-16 | 818 | 226 (27.6%) | 100 (44.2%) | 99 (99.0%) | 1 (1.0%) |
| England | 2016-17 | 421 | 4 (1.0%) | 4 (100%) | - | - |
| England | 2017-18 | 876 | 578 (66.0%) | 578 (100%) | 2 (0.3%) | 576 (99.7%) |
| Guatemala | 2006 | 245 | 2 (0.8%) | 0 (0%) | - | - |
| Guatemala | 2007 | 219 | 1 (0.5%) | 0 (0%) | - | - |
| Guatemala | 2008 | 185 | 63 (34.1%) | 0 (0%) | - | - |
| Guatemala | 2009 | 2267 | 108 (4.8%) | 0 (0%) | - | - |
| Guatemala | 2010 | 584 | 208 (35.6%) | 0 (0%) | - | - |
| Guatemala | 2011 | 285 | 29 (10.2%) | 0 (0%) | - | - |
| Guatemala | 2012 | 416 | 59 (14.2%) | 0 (0%) | - | - |
| Guatemala | 2013 | 212 | 43 (20.3%) | 0 (0%) | - | - |
| Guatemala | 2014 | 109 | 48 (44.0%) | 0 (0%) | - | - |
| Guatemala | 2016 | 210 | 30 (14.3%) | 4 (13.3%) | - | - |
| Guatemala | 2017 | 228 | 54 (23.7%) | 21 (38.9%) | - | - |
| Honduras | 2009 | 904 | 17 (1.9%) | 0 (0%) | - | - |
| Honduras | 2010 | 405 | 78 (19.3%) | 0 (0%) | - | - |
| Honduras | 2011 | 222 | 62 (27.9%) | 0 (0%) | - | - |
| Honduras | 2012 | 142 | 8 (5.6%) | 0 (0%) | - | - |
| Honduras | 2013 | 245 | 50 (20.4%) | 0 (0%) | - | - |
| Honduras | 2014 | 183 | 126 (68.9%) | 0 (0%) | - | - |
| Honduras | 2016 | 127 | 65 (51.2%) | 0 (0%) | - | - |
| Indonesia | 2003 | 112 | 28 (25.0%) | 11 (39.3%) | - | - |
| Indonesia | 2004 | 306 | 98 (32.0%) | 68 (69.4%) | 38 (55.9%) | 30 (44.1%) |
| Indonesia | 2005 | 713 | 385 (54.0%) | 233 (60.5%) | 217 (93.1%) | 16 (6.9%) |
| Indonesia | 2006 | 1041 | 322 (30.9%) | 167 (51.9%) | 96 (57.5%) | 71 (42.5%) |
| Indonesia | 2007 | 1364 | 471 (34.5%) | 182 (38.6%) | 63 (34.6%) | 119 (65.4%) |
| Italy | 2001-02 | 312 | 260 (83.3%) | 0 (0%) | - | - |
| Italy | 2002-03 | 179 | 13 (7.3%) | 0 (0%) | - | - |
| Italy | 2003-04 | 420 | 25 (6.0%) | 0 (0%) | - | - |
| Italy | 2004-05 | 971 | 253 (26.1%) | 0 (0%) | - | - |
| Italy | 2005-06 | 300 | 72 (24.0%) | 0 (0%) | - | - |
| Italy | 2006-07 | 644 | 36 (5.6%) | 0 (0%) | - | - |
| Italy | 2007-08 | 556 | 270 (48.6%) | 0 (0%) | - | - |
| Italy | 2008-09 | 901 | 50 (5.5%) | 7 (14%) | - | - |
| Italy | 2009-10 | 14889 | 69 (0.5%) | 12 (17.4%) | - | - |
| Italy | 2010-11 | 2881 | 794 (27.6%) | 38 (4.8%) | - | - |
| Italy | 2011-12 | 1497 | 58 (3.9%) | 17 (29.3%) | - | - |
| Italy | 2012-13 | 2122 | 1223 (57.6%) | 262 (21.4%) | 8 (3.1%) | 254 (96.9%) |
| Italy | 2013-14 | 1037 | 35 (3.4%) | 8 (22.9%) | - | - |
| Italy | 2014-15 | 3733 | 604 (16.2%) | 262 (43.4%) | 5 (1.9%) | 257 (98.1%) |
| Ivory Coast | 2009 | 321 | 111 (34.6%) | 0 (0%) | - | - |
| Ivory Coast | 2010 | 176 | 62 (35.2%) | 0 (0%) | - | - |
| Ivory Coast | 2011 | 669 | 347 (51.9%) | 0 (0%) | - | - |
| Ivory Coast | 2012 | 246 | 25 (10.2%) | 0 (0%) | - | - |
| Ivory Coast | 2013 | 439 | 153 (34.9%) | 0 (0%) | - | - |
| Ivory Coast | 2014 | 228 | 113 (49.6%) | 92 (81.4%) | 43 (46.7%) | 49 (53.3%) |
| Ivory Coast | 2015 | 335 | 69 (20.6%) | 54 (78.3%) | 7 (13%) | 47 (87%) |
| Ivory Coast | 2016 | 396 | 243 (61.4%) | 224 (92.2%) | 165 (73.7%) | 59 (26.3%) |
| Ivory Coast | 2017 | 375 | 35 (9.3%) | 34 (97.1%) | - | - |
| Kazakhstan | 2010-11 | 238 | 74 (31.1%) | 0 (0%) | - | - |
| Kazakhstan | 2011-12 | 245 | 37 (15.1%) | 0 (0%) | - | - |
| Kazakhstan | 2012-13 | 411 | 163 (39.7%) | 0 (0%) | - | - |
| Kazakhstan | 2013-14 | 301 | 10 (3.3%) | 0 (0%) | - | - |
| Kazakhstan | 2014-15 | 195 | 69 (35.4%) | 4 (5.8%) | - | - |
| Kazakhstan | 2015-16 | 173 | 42 (24.3%) | 0 (0%) | - | - |
| Kazakhstan | 2016-17 | 246 | 95 (38.6%) | 18 (18.9%) | - | - |
| Kenya | 2007 | 641 | 38 (5.9%) | 0 (0%) | - | - |
| Kenya | 2008 | 827 | 220 (26.6%) | 55 (25%) | 3 (5.5%) | 52 (94.5%) |
| Kenya | 2009 | 1640 | 314 (19.1%) | 1 (0.3%) | - | - |
| Kenya | 2010 | 1361 | 174 (12.8%) | 8 (4.6%) | - | - |
| Kenya | 2011 | 1326 | 567 (42.8%) | 73 (12.9%) | 73 (100%) | 0 (0%) |
| Kenya | 2012 | 545 | 177 (32.5%) | 7 (4%) | - | - |
| Kenya | 2013 | 526 | 169 (32.1%) | 0 (0%) | - | - |
| Kenya | 2014 | 664 | 85 (12.8%) | 0 (0%) | - | - |
| Kenya | 2015 | 500 | 137 (27.4%) | 0 (0%) | - | - |
| Kenya | 2016 | 431 | 263 (61.0%) | 0 (0%) | - | - |
| Madagascar | 2005 | 152 | 62 (40.8%) | 49 (79%) | - | - |
| Madagascar | 2006 | 226 | 10 (4.4%) | 10 (100%) | - | - |
| Madagascar | 2007 | 189 | 100 (52.9%) | 100 (100%) | 91 (91%) | 9 (9%) |
| Madagascar | 2008 | 130 | 5 (3.8%) | 5 (100%) | - | - |
| Madagascar | 2009 | 1424 | 265 (18.6%) | 226 (85.3%) | 226 (100%) | 0 (0%) |
| Madagascar | 2010 | 310 | 35 (11.3%) | 17 (48.6%) | - | - |
| Madagascar | 2011 | 472 | 239 (50.6%) | 189 (79.1%) | 189 (100%) | 0 (0%) |
| Madagascar | 2012 | 442 | 224 (50.7%) | 107 (47.8%) | 56 (52.3%) | 51 (47.7%) |
| Madagascar | 2013 | 490 | 285 (58.2%) | 191 (67%) | 0 (0%) | 191 (100%) |
| Madagascar | 2014 | 655 | 194 (29.6%) | 54 (27.8%) | 54 (100%) | 0 (0%) |
| Madagascar | 2015 | 404 | 152 (37.6%) | 98 (64.5%) | 98 (100%) | 0 (0%) |
| Madagascar | 2016 | 375 | 228 (60.8%) | 193 (84.6%) | 193 (100%) | 0 (0%) |
| Morocco | 2004-05 | 187 | 39 (20.9%) | 0 (0%) | - | - |
| Morocco | 2008-09 | 137 | 10 (7.3%) | 0 (0%) | - | - |
| Morocco | 2009-10 | 2121 | 35 (1.7%) | 0 (0%) | - | - |
| Morocco | 2010-11 | 334 | 125 (37.4%) | 0 (0%) | - | - |
| Morocco | 2011-12 | 240 | 16 (6.7%) | 2 (12.5%) | - | - |
| Netherlands | 2005-06 | 136 | 75 (55.1%) | 0 (0.0%) | - | - |
| Netherlands | 2007-08 | 204 | 115 (56.4%) | 0 (0.0%) | - | - |
| Netherlands | 2008-09 | 241 | 51 (21.2%) | 0 (0.0%) | - | - |
| Netherlands | 2009-10 | 363 | 1 (0.3%) | 0 (0.0%) | - | - |
| Netherlands | 2010-11 | 395 | 244 (61.8%) | 177 (72.5%) | 162 (91.5%) | 15 (8.5%) |
| Netherlands | 2011-12 | 122 | 40 (32.8%) | 24 (60%) | - | - |
| Netherlands | 2012-13 | 434 | 244 (56.2%) | 200 (82.0%) | 11 (5.5%) | 189 (94.5%) |
| Netherlands | 2014-15 | 424 | 148 (34.9%) | 121 (81.8%) | 2 (1.7%) | 119 (98.3%) |
| Netherlands | 2015-16 | 472 | 209 (44.3%) | 165 (78.9%) | 158 (95.8%) | 7 (4.2%) |
| Netherlands | 2016-17 | 290 | 35 (12.1%) | 22 (62.9%) | - | - |
| Netherlands | 2017-18 | 612 | 476 (77.8%) | 409 (85.9%) | 5 (1.2%) | 404 (98.8%) |
| New Zealand | 2000 | 303 | 71 (23.4%) | 71 (100%) | 0 (0%) | 71 (100%) |
| New Zealand | 2001 | 640 | 221 (34.5%) | 213 (96.4%) | 0 (0%) | 213 (100%) |
| New Zealand | 2002 | 699 | 149 (21.3%) | 87 (58.4%) | 86 (98.9%) | 1 (1.1%) |
| New Zealand | 2003 | 1108 | 3 (0.3%) | 3 (100%) | - | - |
| New Zealand | 2004 | 864 | 74 (8.6%) | 63 (85.1%) | 1 (1.6%) | 62 (98.4%) |
| New Zealand | 2005 | 845 | 734 (86.9%) | 674 (91.8%) | 550 (81.6%) | 124 (18.4%) |
| New Zealand | 2006 | 768 | 6 (0.8%) | 5 (83.3%) | - | - |
| New Zealand | 2007 | 744 | 163 (21.9%) | 146 (89.6%) | 2 (1.4%) | 144 (98.6%) |
| New Zealand | 2008 | 1053 | 630 (59.8%) | 462 (73.3%) | 354 (76.6%) | 108 (23.4%) |
| New Zealand | 2009 | 4900 | 6 (0.1%) | 0 (0%) | - | - |
| New Zealand | 2010 | 2012 | 10 (0.5%) | 4 (40%) | - | - |
| New Zealand | 2011 | 1268 | 592 (46.7%) | 280 (47.3%) | 276 (98.6%) | 4 (1.4%) |
| New Zealand | 2012 | 2425 | 306 (12.6%) | 120 (39.2%) | 20 (16.7%) | 100 (83.3%) |
| New Zealand | 2013 | 791 | 288 (36.4%) | 256 (88.9%) | 2 (0.8%) | 254 (99.2%) |
| New Zealand | 2014 | 981 | 166 (16.9%) | 117 (70.5%) | 3 (2.6%) | 114 (97.4%) |
| New Zealand | 2015 | 1059 | 479 (45.2%) | 404 (84.3%) | 202 (50%) | 202 (50%) |
| New Zealand | 2016 | 449 | 41 (9.1%) | 31 (75.6%) | - | - |
| New Zealand | 2017 | 1351 | 517 (38.3%) | 453 (87.6%) | 16 (3.5%) | 437 (96.5%) |
| Nicaragua | 2008 | 390 | 152 (39%) | 0 (0%) | - | - |
| Nicaragua | 2009 | 2404 | 0 (0.0%) | - | - | - |
| Nicaragua | 2010 | 646 | 219 (33.9%) | 0 (0%) | - | - |
| Nicaragua | 2011 | 928 | 9 (1.0%) | 0 (0%) | - | - |
| Nicaragua | 2012 | 473 | 352 (74.4%) | 0 (0%) | - | - |
| Nicaragua | 2013 | 939 | 46 (4.9%) | 0 (0%) | - | - |
| Nicaragua | 2014 | 989 | 567 (57.3%) | 18 (3.2%) | - | - |
| Nicaragua | 2015 | 362 | 0 (0.0%) | - | - | - |
| Nicaragua | 2016 | 267 | 69 (25.8%) | 0 (0%) | - | - |
| Nicaragua | 2017 | 762 | 428 (56.2%) | 0 (0%) | - | - |
| Panama | 2008 | 119 | 27 (22.7%) | 0 (0%) | - | - |
| Panama | 2009 | 1270 | 81 (6.4%) | 0 (0%) | - | - |
| Panama | 2010 | 328 | 56 (17.1%) | 0 (0%) | - | - |
| Panama | 2012 | 269 | 177 (65.8%) | 0 (0%) | - | - |
| Panama | 2013 | 188 | 0 (0.0%) | - | - | - |
| Panama | 2014 | 141 | 55 (39%) | 55 (100%) | 0 (0%) | 55 (100%) |
| Panama | 2016 | 810 | 4 (0.5%) | 4 (100%) | - | - |
| Panama | 2017 | 262 | 141 (53.8%) | 141 (100%) | 85 (60.3%) | 56 (39.7%) |
| Portugal | 2001-02 | 143 | 6 (4.2%) | 0 (0%) | - | - |
| Portugal | 2003-04 | 150 | 0 (0.0%) | - | - | - |
| Portugal | 2004-05 | 192 | 25 (13.0%) | 0 (0%) | - | - |
| Portugal | 2008-09 | 156 | 8 (5.1%) | 0 (0%) | - | - |
| Portugal | 2009-10 | 113 | 6 (5.3%) | 3 (50%) | - | - |
| Portugal | 2010-11 | 131 | 34 (26.0%) | 34 (100%) | - | - |
| Portugal | 2013-14 | 467 | 7 (1.5%) | 7 (100%) | - | - |
| Portugal | 2014-15 | 498 | 328 (65.9%) | 328 (100%) | 0 (0%) | 328 (100%) |
| Portugal | 2015-16 | 449 | 37 (8.2%) | 37 (100%) | - | - |
| Portugal | 2016-17 | 473 | 1 (0.2%) | 1 (100%) | - | - |
| Singapore | 2007 | 325 | 257 (79.1%) | 0 (0%) | - | - |
| Singapore | 2008 | 763 | 349 (45.7%) | 0 (0%) | - | - |
| Singapore | 2009 | 5131 | 151 (2.9%) | 0 (0%) | - | - |
| Singapore | 2010 | 3563 | 785 (22%) | 417 (53.1%) | 398 (95.4%) | 19 (4.6%) |
| Singapore | 2011 | 1201 | 286 (23.8%) | 156 (54.5%) | 119 (76.3%) | 37 (23.7%) |
| Singapore | 2012 | 1007 | 482 (47.9%) | 331 (68.7%) | 190 (57.4%) | 141 (42.6%) |
| Singapore | 2013 | 770 | 172 (22.3%) | 166 (96.5%) | 56 (33.7%) | 110 (66.3%) |
| Singapore | 2014 | 1002 | 390 (38.9%) | 327 (83.8%) | 15 (4.6%) | 312 (95.4%) |
| Singapore | 2015 | 790 | 140 (17.7%) | 136 (97.1%) | 27 (19.9%) | 109 (80.1%) |
| Singapore | 2016 | 1181 | 415 (35.1%) | 364 (87.7%) | 235 (64.6%) | 129 (35.4%) |
| Singapore | 2017 | 998 | 306 (30.7%) | 263 (85.9%) | 78 (29.7%) | 185 (70.3%) |
| South Africa | 2009 | 437 | 27 (6.2%) | 3 (11.1%) | - | - |
| South Africa | 2010 | 326 | 199 (61.0%) | 86 (43.2%) | 78 (90.7%) | 8 (9.3%) |
| South Africa | 2011 | 466 | 179 (38.4%) | 86 (48%) | 38 (44.2%) | 48 (55.8%) |
| South Africa | 2012 | 287 | 156 (54.4%) | 118 (75.6%) | 99 (83.9%) | 19 (16.1%) |
| South Africa | 2013 | 174 | 31 (17.8%) | 23 (74.2%) | - | - |
| South Africa | 2015 | 176 | 45 (25.6%) | 17 (37.8%) | - | - |
| South Africa | 2016 | 232 | 108 (46.6%) | 106 (98.1%) | 48 (45.3%) | 58 (54.7%) |
| South Africa | 2017 | 220 | 70 (31.8%) | 59 (84.3%) | 3 (5.1%) | 56 (94.9%) |
| Turkey | 2007-08 | 144 | 52 (36.1%) | 52 (100%) | 0 (0%) | 52 (100%) |
| Turkey | 2009-10 | 1280 | 0 (0.0%) | - | - | - |
| Turkey | 2010-11 | 566 | 302 (53.4%) | 302 (100%) | 0 (0%) | 302 (100%) |
| Ukraine | 2002-03 | 100 | 0 (0.0%) | - | - | - |
| Ukraine | 2006-07 | 105 | 27 (25.7%) | 27 (100%) | - | - |
| Ukraine | 2007-08 | 229 | 64 (27.9%) | 23 (35.9%) | - | - |
| Ukraine | 2008-09 | 112 | 22 (19.6%) | 22 (100%) | - | - |
| Ukraine | 2011-12 | 184 | 7 (3.8%) | 6 (85.7%) | - | - |
| Ukraine | 2012-13 | 336 | 70 (20.8%) | 50 (71.4%) | 0 (0%) | 50 (100%) |
| Ukraine | 2013-14 | 185 | 15 (8.1%) | 8 (53.3%) | - | - |
| Ukraine | 2014-15 | 284 | 183 (64.4%) | 29 (15.8%) | - | - |
| Ukraine | 2015-16 | 399 | 1 (0.3%) | 0 (0%) | - | - |
| USA | 2000-01 | 10506 | 4909 (46.7%) | 0 (0%) | - | - |
| USA | 2001-02 | 16402 | 2230 (13.6%) | 0 (0%) | - | - |
| USA | 2002-03 | 9811 | 4083 (41.6%) | 0 (0%) | - | - |
| USA | 2003-04 | 25088 | 289 (1.2%) | 0 (0%) | - | - |
| USA | 2004-05 | 24568 | 6199 (25.2%) | 0 (0%) | - | - |
| USA | 2005-06 | 21230 | 4398 (20.7%) | 0 (0%) | - | - |
| USA | 2006-07 | 24964 | 5328 (21.3%) | 0 (0%) | - | - |
| USA | 2007-08 | 42390 | 12214 (28.8%) | 0 (0%) | - | - |
| USA | 2008-09 | 74575 | 11261 (15.1%) | 0 (0%) | - | - |
| USA | 2009-10 | 110176 | 441 (0.4%) | 0 (0%) | - | - |
| USA | 2010-11 | 56143 | 14642 (26.1%) | 0 (0%) | - | - |
| USA | 2011-12 | 25689 | 4500 (17.5%) | 0 (0%) | - | - |
| USA | 2012-13 | 80406 | 23952 (29.8%) | 0 (0%) | - | - |
| USA | 2013-14 | 58553 | 8348 (14.3%) | 0 (0%) | - | - |
| USA | 2014-15 | 128925 | 21930 (17.0%) | 0 (0%) | - | - |
| USA | 2015-16 | 99381 | 31880 (32.1%) | 0 (0%) | - | - |
| USA | 2016-17 | 175150 | 49398 (28.2%) | 0 (0%) | - | - |
| Viet Nam | 2006 | 947 | 315 (33.3%) | 0 (0%) | - | - |
| Viet Nam | 2007 | 1169 | 274 (23.4%) | 0 (0%) | - | - |
| Viet Nam | 2008 | 1488 | 620 (41.7%) | 0 (0%) | - | - |
| Viet Nam | 2009 | 1931 | 447 (23.1%) | 0 (0%) | - | - |
| Viet Nam | 2010 | 962 | 495 (51.5%) | 0 (0%) | - | - |
| Viet Nam | 2011 | 666 | 183 (27.5%) | 0 (0%) | - | - |
| Viet Nam | 2012 | 674 | 398 (59.1%) | 0 (0%) | - | - |
| Viet Nam | 2013 | 775 | 254 (32.8%) | 0 (0%) | - | - |
| Viet Nam | 2017 | 230 | 103 (44.8%) | 0 (0%) | - | - |
