# Supplementary material for: The epidemiological signature of influenza B virus and its B/Victoria and B/Yamagata lineages in the 21st century
Source: PLoS One. 2019 Sep 12;14(9):e0222381. doi: 10.1371/journal.pone.0222381 (PMC6742362; doi:10.1371/journal.pone.0222381)

Madagascar (median age 19.7 years)

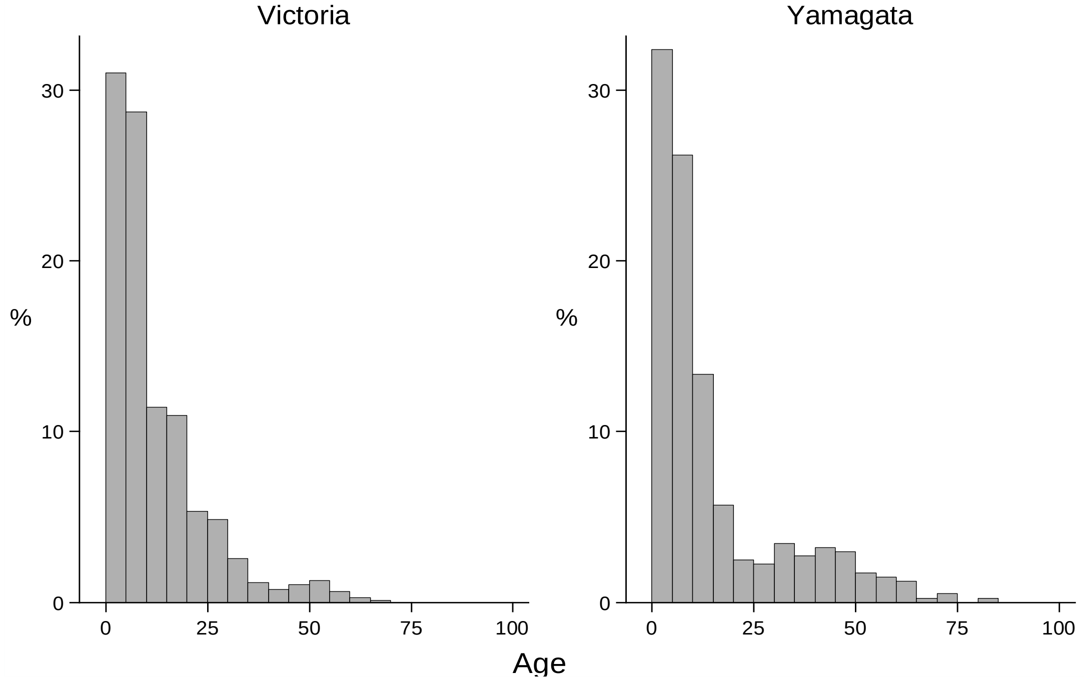

Ivory Coast (median age 20.9 years)

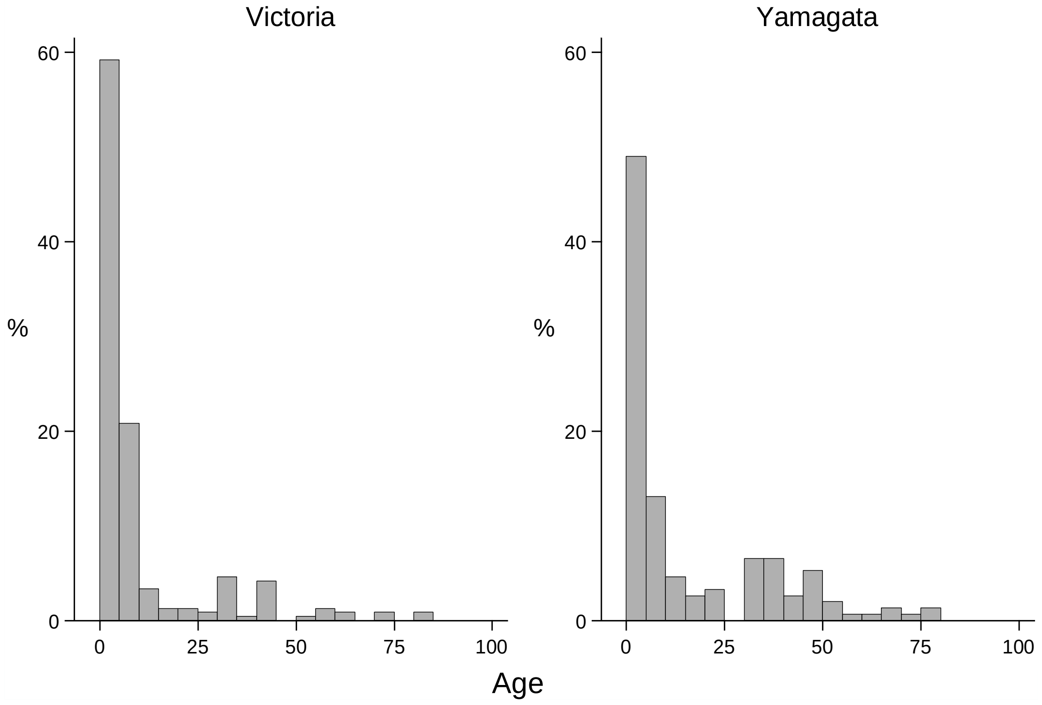

South Africa (median age 27.1 years)

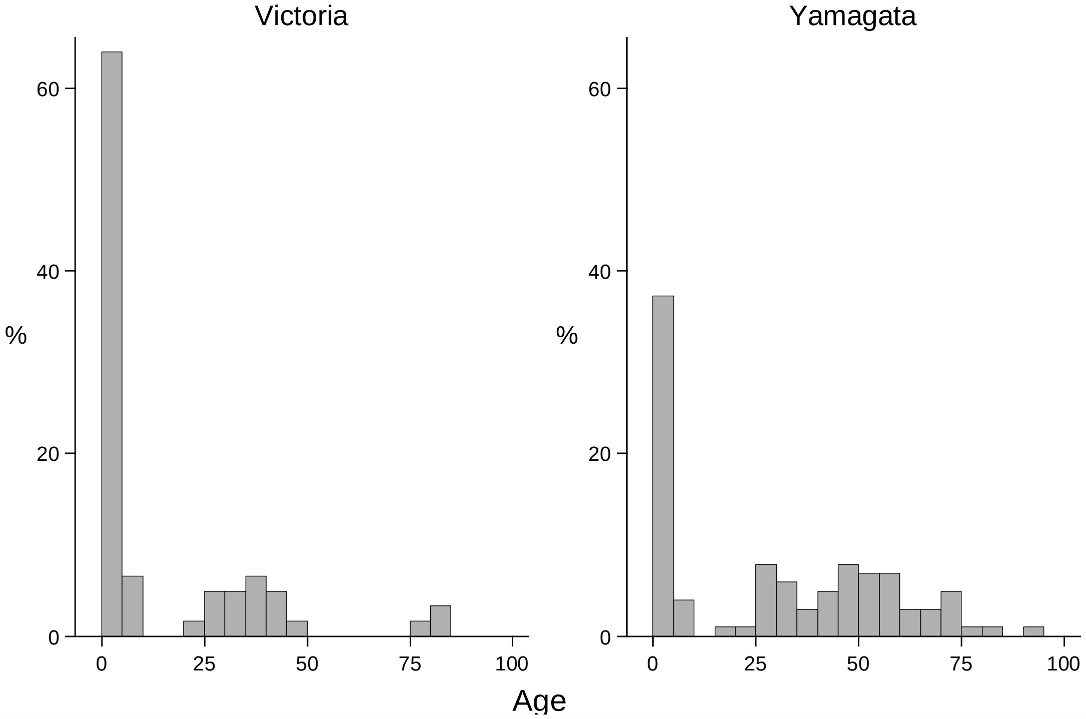

Indonesia (median age 30.2 years)

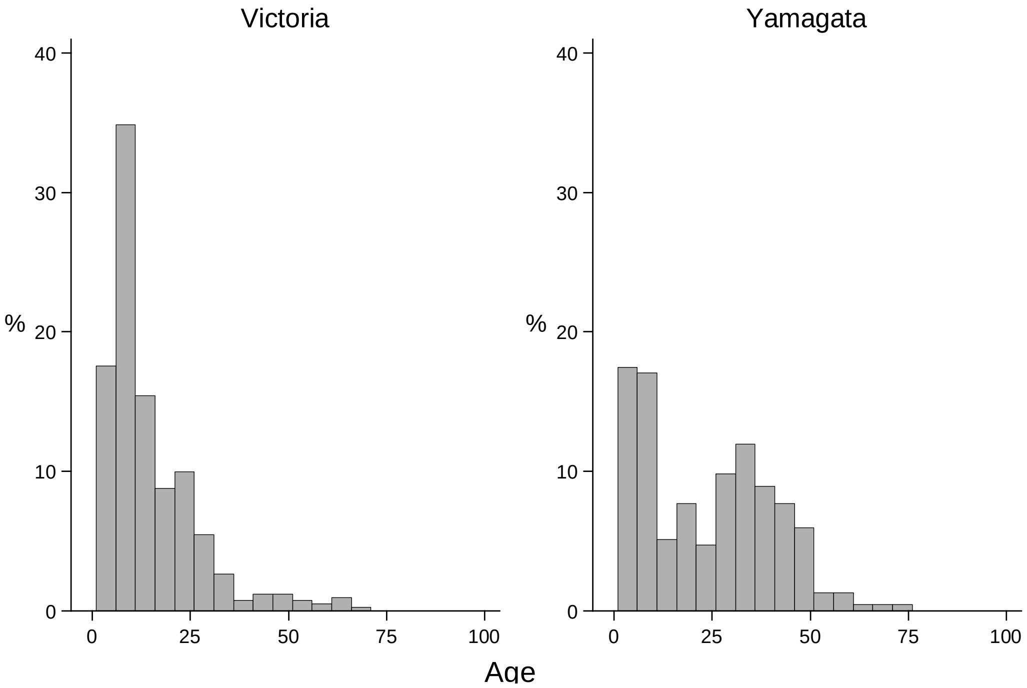

Turkey (median age 30.9 years)

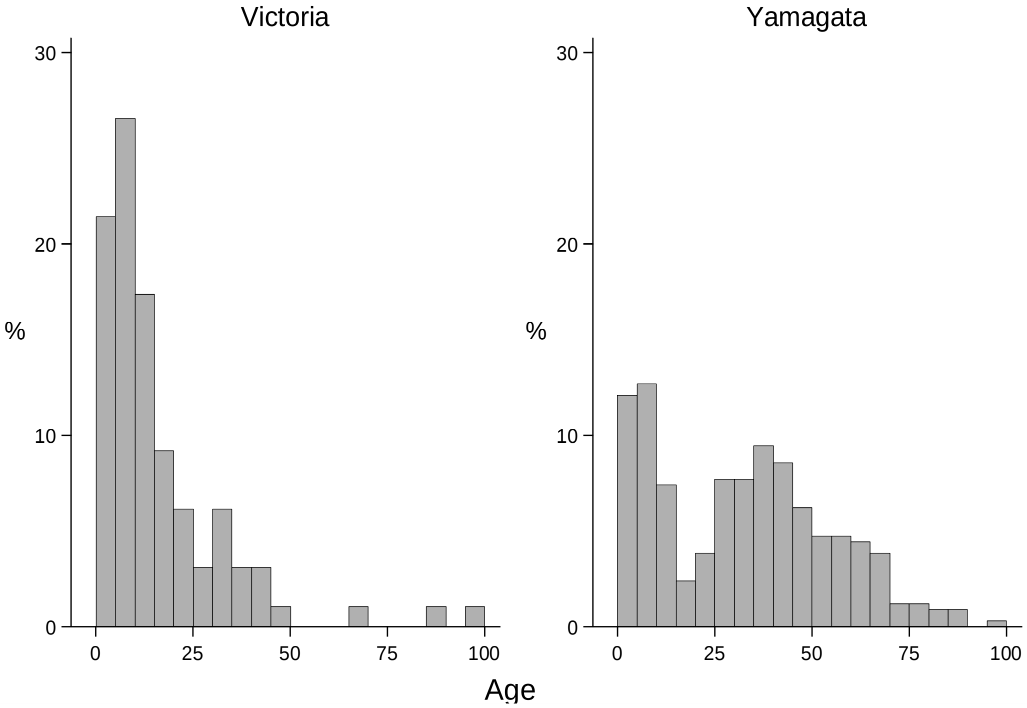

Chile (median age 34.4 years)

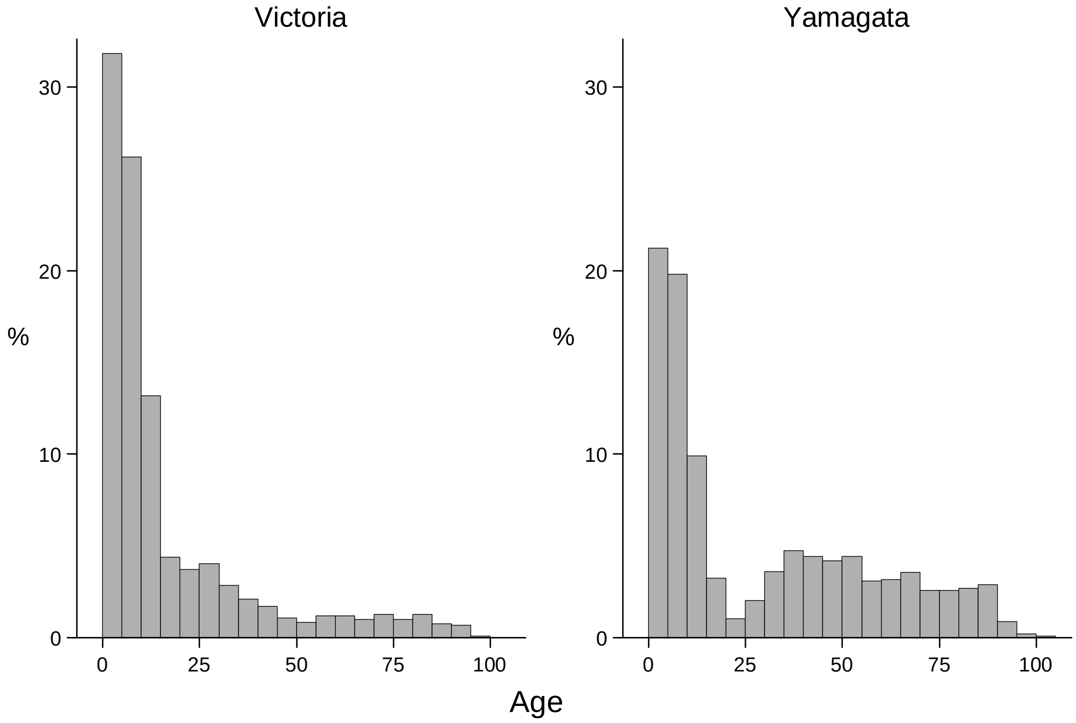

Singapore (median age 34.6 years)

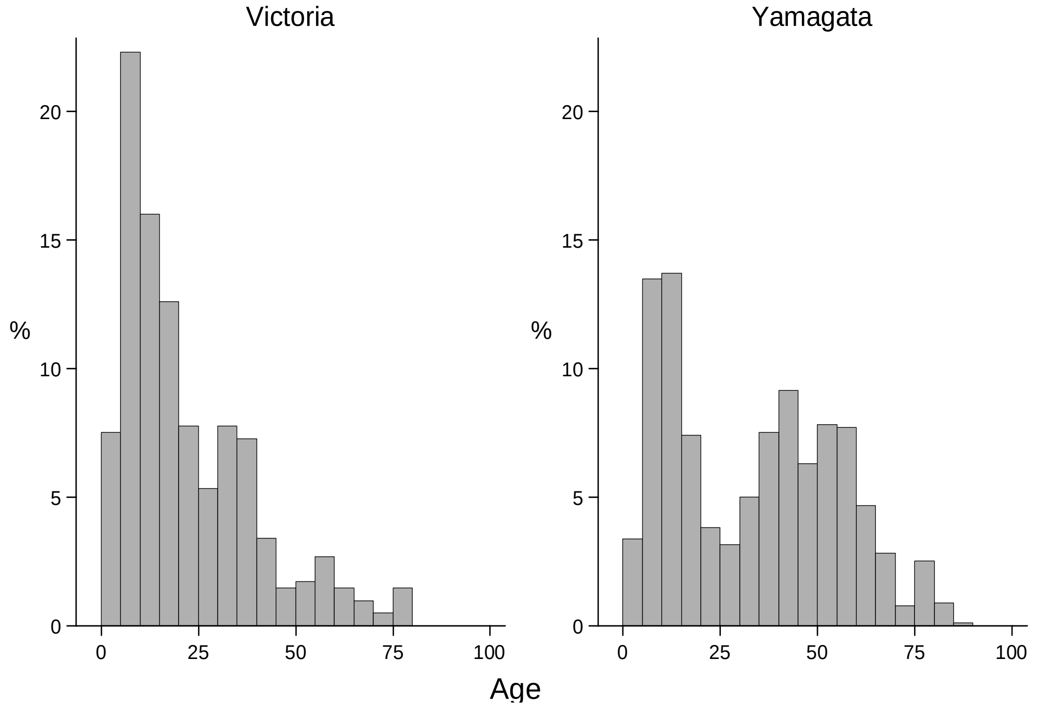

New Zealand (median age 37.9 years)

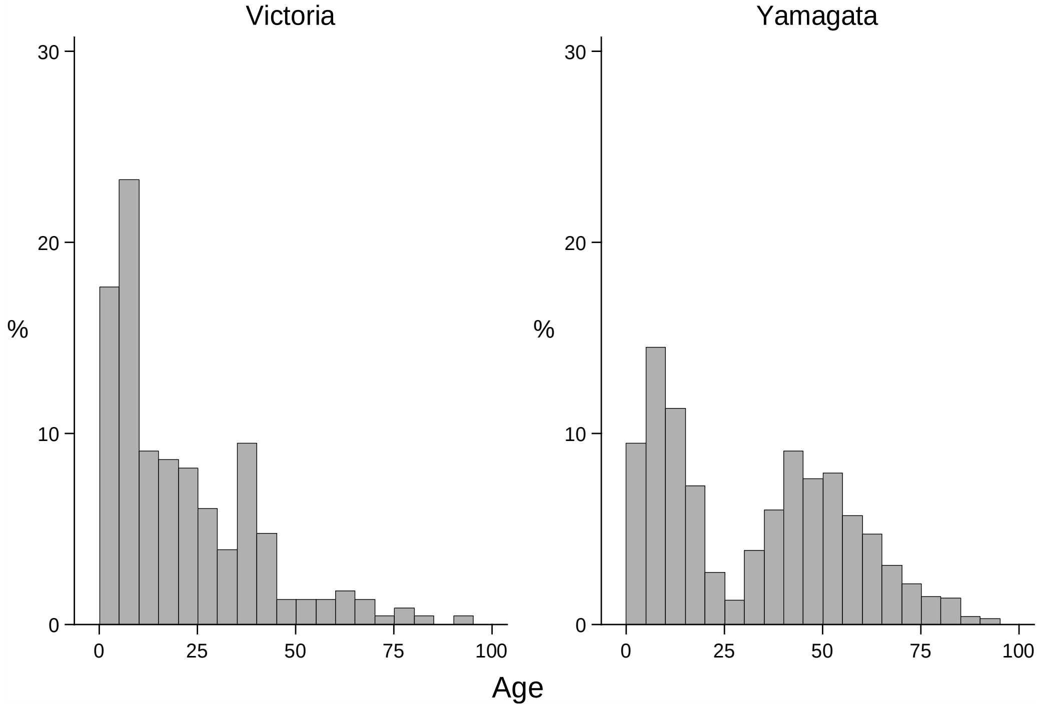

Australia (median age 38.7 years)

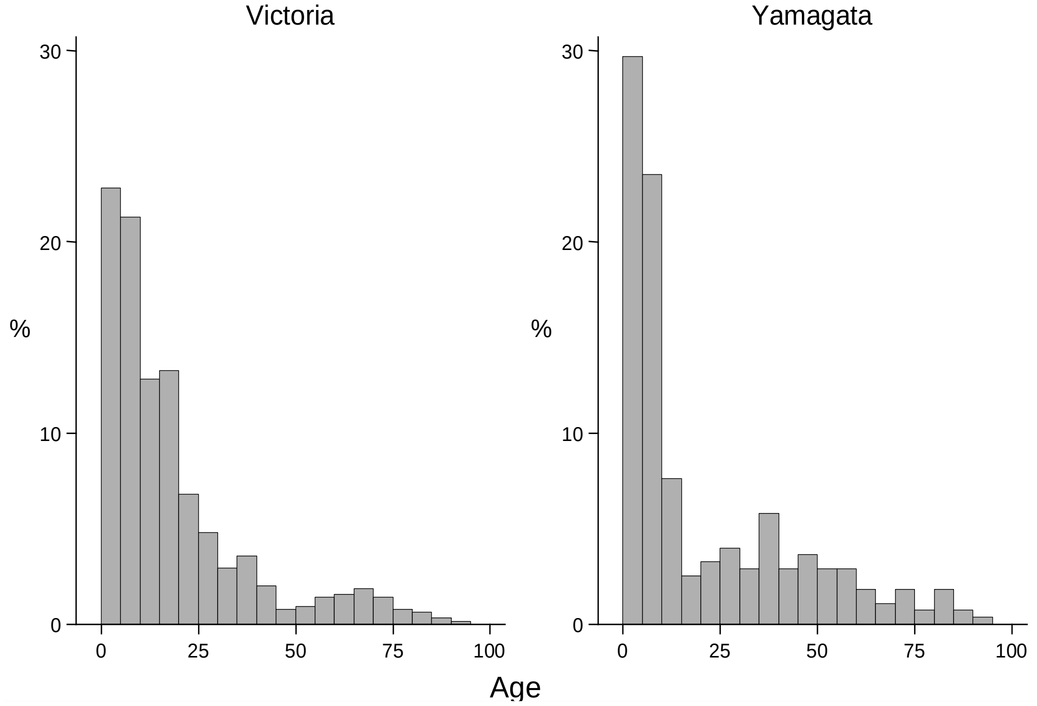

England (median age 40.5 years)

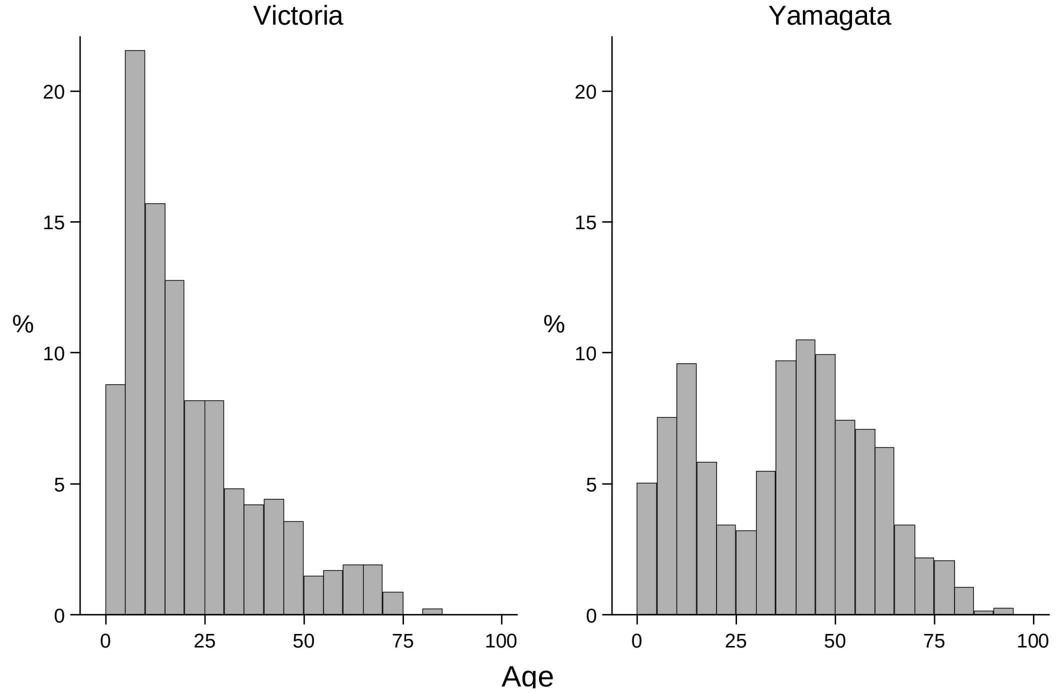

Ukraine (median age 40.6 years)

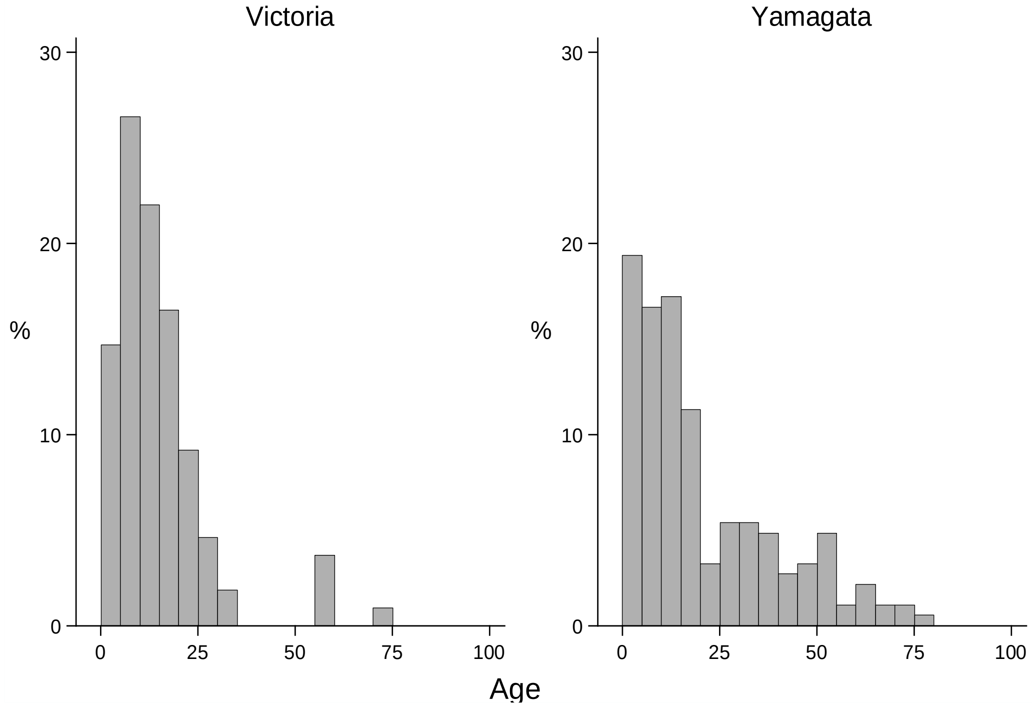

Portugal (median age 42.2 years)

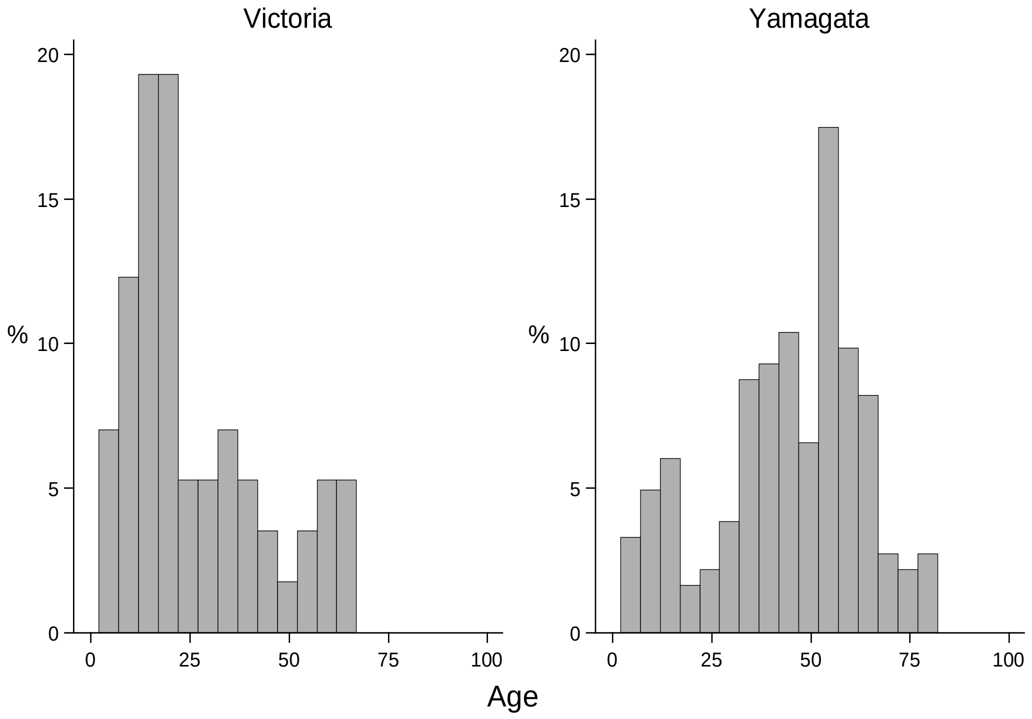

Netherlands (median age 42.6 years)

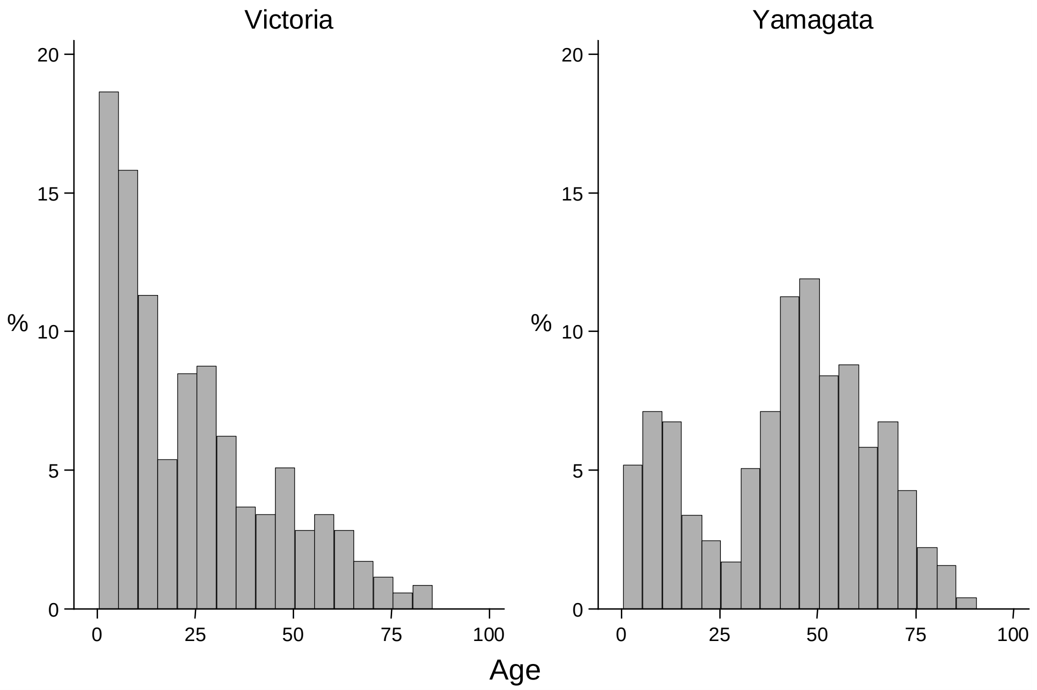

Italy (median age 45.5 years)

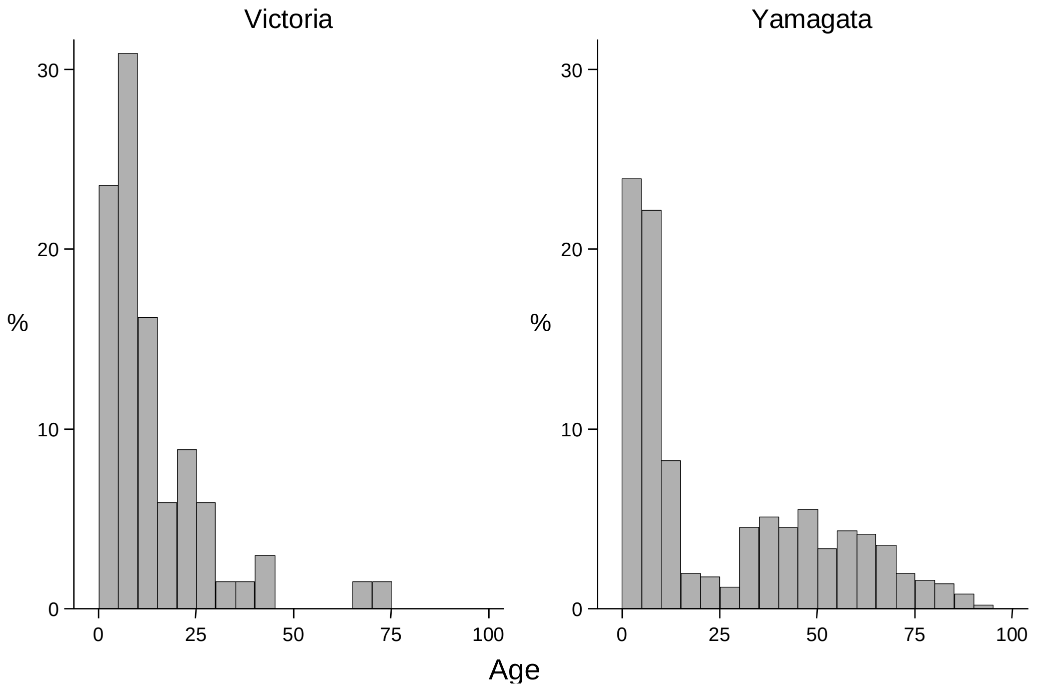

Supplement: S1 File — Countries which provided information on exact age for ≥50 B Victoria and ≥50 B Yamagata influenza for the whole study period cases were included. Countries were ordered according to the median age of the general population. The Global Influenza B Study, 2000–2018. (PDF) [file pone.0222381.s002.pdf]
