# Supplementary material for: The epidemiological signature of influenza B virus and its B/Victoria and B/Yamagata lineages in the 21st century
Source: PLoS One. 2019 Sep 12;14(9):e0222381. doi: 10.1371/journal.pone.0222381 (PMC6742362; doi:10.1371/journal.pone.0222381)
Supplement: S2 File — (DOC) [file pone.0222381.s003.doc]

The database of the Global Influenza B Study was created by collecting surveillance datasets from each of the participating countries. These national datasets are owned by the participating countries and thus cannot be shared publicly by the study group at NIVEL. Researchers interested in accessing the complete GIBS database can contact John Paget ([j.paget@nivel.nl](mailto:j.paget@nivel.nl)) or Saverio Caini ([s.caini@nivel.nl](mailto:s.caini@nivel.nl)): any request to access the GIBS database will need, however, to be approved by the study group at NIVEL and by contributors from each participating country. Researchers interested in obtaining the country-specific datasets may instead contact the individuals listed below:

Argentina (Santa Fe Province): Gabriela Kusznierz (labconi@yahoo.com.ar)

Australia: publically available data were obtained from the website of the Australia Influenza

Surveillance Network.

Bhutan: Sonam Wangchuk (swangchuk@health.gov.bt)

Brazil: Francisco José de Paula Júnior (francisco.pjunior@saude.gov.br)

Cameroon: Richard Njouom (njouom@yahoo.com)

Chile: Rodrigo Fasce (rfasce@ispch.cl)

China: Feng Luzhao (fenglz@chinacdc.cn)

Costa Rica, El Salvador, Guatemala, Honduras, Nicaragua, Panama: Rakhee Palekar

(palekarr@paho.org)

Ecuador: Alfredo Bruno (alfredobruno@yahoo.es)

England: Maria Zambon (maria.zambon@phe.gov.uk)

Indonesia: Herman Kosasih (herman_kosasih@yahoo.com)

Italy: Caterina Rizzo (caterina.rizzo@iss.it)

Ivory Coast: Herve A. Kadjo (rvkdjo@yahoo.fr)

Kazakhstan: Ainash Makusheva (stamm_csee_astana11@mail.ru)

Kenya: J Sandra S. Chaves (bev8@cdc.gov)

Madagascar: Jean-Michel Heraud (jmheraud@pasteur.mg)

Morocco: Amal Barakat (amal.barakat@yahoo.fr)

Netherlands: Adam Meijer (adam.meijer@rivm.nl)

New Zealand: Sue Huang (sue.huang@esr.cri.nz)

Portugal: Ana Paula Rodrigues (ana.rodrigues@insa.min-saude.pt)

Singapore: Vernon Jian Ming Lee (vernon_lee@moh.gov.sg)

South Africa: Cheryl Cohen (cherylc@nicd.ac.za)

Turkey: Meral Akcay Ciblak (ciblakm@yahoo.com)

Ukraine: Alla Mironenko (miralla@ukr.net)

USA: Joseph Bresee (jbresee@cdc.gov)

Viet Nam: Mai T.Q. Le (lom9@hotmail.com)
